# Development of virus-like particles with inbuilt immunostimulatory properties as vaccine candidates

**DOI:** 10.3389/fmicb.2023.1065609

**Published:** 2023-06-07

**Authors:** Simon Collett, Linda Earnest, Julio Carrera Montoya, Melissa A. Edeling, Ashley Yap, Chinn Yi Wong, Dale Christiansen, Jason Roberts, Jamie Mumford, Valerie Lecouturier, Vincent Pavot, Sergio Marco, Joon Keit Loi, Cameron Simmons, Shivali A. Gulab, Jason M. Mackenzie, Aaron Elbourne, Paul A. Ramsland, Garth Cameron, Dhiraj Hans, Dale I. Godfrey, Joseph Torresi

**Affiliations:** ^1^School of Science, College of Science, Engineering and Health, RMIT University, Melbourne, VIC, Australia; ^2^Department of Microbiology and Immunology, The Peter Doherty Institute for Infection and Immunity, University of Melbourne, Parkville, VIC, Australia; ^3^Victorian Infectious Diseases Reference Laboratory, Royal Melbourne Hospital at the Doherty Institute for Infection and Immunity, Melbourne, VIC, Australia; ^4^Department of Infectious Diseases, The University of Melbourne at the Doherty Institute for Infection and Immunity, Melbourne, VIC, Australia; ^5^Sanofi-Pasteur, Lyon, France; ^6^Institute of Vector-Borne Disease, Monash University, Clayton, VIC, Australia; ^7^Avalia Immunotherapies Limited, Wellington, New Zealand; ^8^Vaccine Alliance Aotearoa New Zealand, Wellington, New Zealand; ^9^Department of Surgery Austin Health, University of Melbourne, Heidelberg, VIC, Australia; ^10^Department of Immunology, Central Clinical School, Monash University, Melbourne, VIC, Australia; ^11^Research, Innovation and Commercialisation, Faculty of Medicine, Dentistry and Health Sciences, University of Melbourne, Parkville, VIC, Australia

**Keywords:** flavivirus, arbovirus, adjuvant, VLP, immunology, SARS-CoV-2, hepatitis, vaccine platform

## Abstract

The development of virus-like particle (VLP) based vaccines for human papillomavirus, hepatitis B and hepatitis E viruses represented a breakthrough in vaccine development. However, for dengue and COVID-19, technical complications, such as an incomplete understanding of the requirements for protective immunity, but also limitations in processes to manufacture VLP vaccines for enveloped viruses to large scale, have hampered VLP vaccine development. Selecting the right adjuvant is also an important consideration to ensure that a VLP vaccine induces protective antibody and T cell responses. For diseases like COVID-19 and dengue fever caused by RNA viruses that exist as families of viral variants with the potential to escape vaccine-induced immunity, the development of more efficacious vaccines is also necessary. Here, we describe the development and characterisation of novel VLP vaccine candidates using SARS-CoV-2 and dengue virus (DENV), containing the major viral structural proteins, as protypes for a novel approach to produce VLP vaccines. The VLPs were characterised by Western immunoblot, enzyme immunoassay, electron and atomic force microscopy, and *in vitro* and *in vivo* immunogenicity studies. Microscopy techniques showed proteins self-assemble to form VLPs authentic to native viruses. The inclusion of the glycolipid adjuvant, α-galactosylceramide (α-GalCer) in the vaccine formulation led to high levels of natural killer T (NKT) cell stimulation *in vitro*, and strong antibody and memory CD8^+^ T cell responses *in vivo*, demonstrated with SARS-CoV-2, hepatitis C virus (HCV) and DEN VLPs. This study shows our unique vaccine formulation presents a promising, and much needed, new vaccine platform in the fight against infections caused by enveloped RNA viruses.

## 1. Introduction

Vaccines are amongst the most effective preventative interventions in medicine. The most effective vaccines in clinical use include attenuated viral, inactivated whole virus, subunit, synthetic conjugate and more recently mRNA vaccines (Carreno et al., 2014; Rauch et al., 2018; Vetter et al., 2018; Mascola and Fauci, 2020). Virus-like particles (VLPs) also offer effective vaccine candidates because they resemble the mature parent virus and present antigens in a repetitive manner, inducing both protective humoral and cellular immune responses. In addition, VLPs can rapidly and efficiently traffic from the primary site of inoculation to regional lymph nodes to potently drive vaccine specific immune responses (Cubas et al., 2009; Qian et al., 2020). The long-term safety and efficacy profile of commercial VLP-based vaccines for viruses like hepatitis B, human papilloma and hepatitis E viruses (Zhang et al., 2015; Patel et al., 2018; Liu et al., 2022) attests to an approach for the development of VLP vaccines for infections like Severe Acute Respiratory Syndrome Coronavirus-2 (SARS-CoV-2), dengue (DENV) and other respiratory viruses. For SARS-CoV-2, VLPs may also provide a safer and more immunologically durable alternative to SARS-CoV-2 mRNA vaccines.

Manufacturing VLP vaccines is limited by the difficulty in co-expressing viral structural proteins in cell culture in a manner to allow assembly and production of large quantities of VLPs. For some viruses, like influenza, the structural proteins are produced from individual gene segments. For others like DENV, the structural proteins [capsid (C), pre-membrane (prM), and envelope (E)] are cleaved from a single polyprotein by a combination of the viral NS2B-3 protease and host cell peptidase. In contrast, the structural proteins of hepatitis C virus (HCV), which are also produced as a polyprotein, are cleaved by cellular signal peptidase (SP) followed by a unique second signal peptide peptidase (SPP) cleavage which is essential for the release of the core protein from the endoplasmic reticulum (ER) and subsequent particle assembly (Lemberg and Martoglio, 2002; McLauchlan et al., 2002; Pene et al., 2009; Oehler et al., 2012). Previously, we took advantage of the unique nature of SPP cleavage in the ER to develop a quadrivalent HCV vaccine from a single SP/SPP regulated (self) cleaving polyprotein containing the core and envelope proteins. This strategy led to the production of quadrivalent HCV VLPs that we previously showed to be strongly immunogenic, inducing HCV specific neutralising antibody (NAb) and T cell responses (Earnest-Silveira et al., 2016a,b; Kumar et al., 2016; Christiansen et al., 2018a,b, 2019). We then adapted this method for the production of VLPs derived from other viruses, including DENV and SARS-CoV-2.

COVID-19 has presented the greatest global health challenge in over 100 years with over 685 million infections, 6.85 million deaths and impact across 200 countries. The continued resurgence of COVID-19 is fueled by the emergence of SARS-CoV-2 variants which are better adapted at escaping protective immune responses and at infecting humans (Kurhade et al., 2022; Qu et al., 2022; Suryawanshi et al., 2022; Takashita et al., 2022; Tegally et al., 2022). Current COVID-19 vaccines have relied mainly on the delivery of the spike (S) protein or gene to induce S-specific immune responses, including receptor binding domain (RBD) NAb responses (Corbett et al., 2020; Sahin et al., 2020; Ewer et al., 2021). These have proven to be highly effective (Polack et al., 2020; Dagan et al., 2021; Hall et al., 2021; Vasileiou et al., 2021; Voysey et al., 2021). However, the emergence of Omicron and its many sublineages has threatened the long-term efficacy of these vaccines (Kurhade et al., 2022; Qu et al., 2022; Takashita et al., 2022; Torresi et al., 2022).

DENV is also one of the world’s major public health threats causing an estimated 96 million symptomatic infections, 500,000 hospitalisations and 25,000 deaths each year (Bhatt et al., 2013). Currently, more than half the world’s population (approximately 4.7 billion people) live in endemic regions of tropical and subtropical climates, particularly in South and South-East Asia, Africa, and South and Central America. A major challenge has been how to design a vaccine that is able to induce cross protective immunity to all 4 serotypes of DENV without the risk of causing more severe DENV infection in vaccine recipients, through antibody-dependent enhancement (ADE) (Guzman and Vazquez, 2010; Halstead et al., 2010). Broadly neutralising antibodies provide serotype-specific cross-protective responses against DENV (Lok et al., 2008; Fibriansah et al., 2013; Dejnirattisai et al., 2015; Gallichotte et al., 2015). Recent work has shown that VLPs composed of the prM and E proteins of DENV serotypes 1–4 present many conformational and quaternary structure-dependent epitopes including the envelope protein domain 3 (EDIII) that are recognised by well-characterised anti-DENV NAbs (Lazo et al., 2007; Beltramello et al., 2010; Metz et al., 2018; Rajpoot et al., 2018; Ramasamy et al., 2018; Utomo et al., 2020; Imagawa et al., 2021).

However, the ability of VLP vaccines to stimulate potent protective and long-term memory immune responses may be limited. To overcome this issue, immunostimulatory adjuvants are often delivered along with vaccines. Integrating an adjuvant directly into a VLP vaccine would not only be a major advance for vaccine immunogenicity but also greatly facilitate manufacturing. Glycolipids, such as alpha-galactosyl ceramide (α-GalCer), have the potential to fulfil this requirement (Carreno et al., 2014). α-GalCer has gained considerable interest as a potential vaccine adjuvant because it is able to recruit NKT cells that stimulate the development of antibody, cytotoxic and memory T cell responses (Guillonneau et al., 2009; Godfrey et al., 2015; Anderson et al., 2017; Liu and Guo, 2017; Holz et al., 2020). Activated NKT cells essentially provide a source of universal T cell help to activate an array of immune cell types including dendritic cells, B cells and conventional T cells (Godfrey et al., 2015).

In this report, we developed novel VLPs and herein describe their production, characterisation, and immunogenicity. Additionally, we showed that α-GalCer can be incorporated into the VLPs of different RNA viruses during particle production and that this results in enhanced immune responses. Our method allows for large-scale laboratory production of VLP-based vaccines that we have adapted for enveloped RNA viruses, including HCV, SARS-CoV-2 and DENV and that can be applied to developing potent self-adjuvanted multivalent viral vaccines. This study uses VLPs from the three viruses to investigate different aspects of production, characterisation, and analysis of the immune response to the VLPs, as proof of principle.

## 2. Methods and materials

### 2.1. Production and purification of SARS-CoV-2 and DEN VLPs

A synthetic SARS-CoV-2 gene construct of 4.8 kb (NCBI Reference Sequence: NC_045512.2) that encodes a polyprotein of the structural proteins Spike (3.831 kb), Envelope (225 bp) and Membrane (680 bp) (GeneArt, Thermofisher Scientific, United States) was synthesised with HCV core signal peptide between the S, E and M gene. The construct supplied was resuspended in sterile water to a concentration of 0.25 μg/μL. One microlitre of gene construct was transformed into Top 10F’ bacterial competent cells and plated onto Luria Broth agar plates with Kanamycin (50 μg/mL) and plates incubated overnight at 37°C. Colonies were then picked and used to inoculate 5 mL LB-Kan (50 μg/mL). After overnight growth at 37°C with shaking at 180 rpm, plasmid DNA was extracted by Qiagen Plasmid Mini Kit (12123, Qiagen, Germany). The 4.8 kb SARS-CoV-2 DNA was released by digesting with *Kpn*I (R3142, NEB, United States) and *Not*I-HF (R3189S, NEB, United States) and ligated into the vector pAdTrackCMV using T4 DNA Ligase (M0202T, NEB, United States).

The reaction was incubated at 16°C for 2 h and then transformed into chemically competent Top10F’ cells using standard competent cell transformation protocols. Cells were then plated onto LB Agar Kanamycin (50 μg/mL) plates and incubated at 37°C overnight. Colonies were selected, grown overnight, plasmid extracted and inserts confirmed by digestion with *Kpn*I and *Not*I. Positive clones were sent for DNA sequence confirmation to the Australian Genome Research Facility (AGRF, WEHI, Parkville, Victoria). One confirmed the clone was then linearized with *Pme*I (R0560S, NEB, United States), phenol-chloroform extracted and ethanol precipitated overnight at −80°C. The following day, tubes were spun down, the DNA pellet washed with 70% ethanol and air-dried. After resuspending the pellet in nuclease-free water, DNA was quantitated on a Nanodrop 2000 (Thermofisher Scientific, Germany) and recombined with BJ5183-AD-1 Electroporation Competent Cells (Adeasier Cells, 200157, Agilent USA) using a BioRad Micropulser Electroporator. Potential colonies were selected and grown overnight in LB Kanamycin (50 μg/mL). Plasmid DNA was extracted using the Qiagen Plasmid Mini kit and digested with *Pac*I. Correct recombinants that yield a large fragment (approximately 30 kb) and a smaller fragment of 3.0 or 4.5 kb, were then selected to be transformed into Top10F’ competent cells for stability. Colonies were selected the following day and grown up overnight for extraction of the Adeno SARS-CoV-2 construct using the Qiagen Plasmid Maxi Kit (12162, Qiagen, Germany). Positive clones were again sent for DNA sequence confirmation to the Australian Genome Research Facility (AGRF, WEHI). Digestion of the selected plasmid was then set up with *Pac*I (R0547S, NEB, United States), ethanol precipitated and quantitated for transfection into 293A cells.

DEN VLPs containing structural proteins of DENV of serotypes 2 (M84727.1 Dengue virus type 2 strain 16681), 3 (KF955491 Dengue virus 3 isolate DENV-3/NI/BID-V3074/2008) and 4 (KY586894 Dengue virus isolate Ser4_Thailand_Bangkok_Seq43) were produced using cell culture methods as previously described for the large-scale production of a quadrivalent HCV VLP-based vaccine candidate (Kumar et al., 2016; Christiansen et al., 2018a,b, 2019). Herein, recombinant adenovirus constructs encoding the DENV structural proteins [capsid (C, 356 bp), precursor membrane (prM, 272 bp) and envelope (E, 1484 bp) proteins] were developed for DENV serotypes 2, 3 and 4, including the following cloning modifications: For each serotype, constructs were generated with modifications to ensure proper processing and secretion of viral proteins after transduction of mammalian cells. The DENV capsid protein cleavage sequence was replaced by a sequence that is cleaved by an ER SPP (Oehler et al., 2012). Additionally, the DENV E protein ER retention signal was altered by introducing three non-synonymous point mutations into the E stem anchor (I398L, M401A and M412L substitutions) to enhance the extracellular secretion of the E protein (clone rAd-DENcapsid_SPP_-prM/E-3ptmut) (Purdy and Chang, 2005).

The DENcapsid_SPP_-prM/E-3ptmut genome was synthesised in the pBHK vector (Bioneer Pacific) and subcloned into pAdTrack CMV. The pAdTrack-CMV-DEN2capsid_SPP_-prM/E-3ptmut plasmid was digested with *Pme*I and transformed into chemically competent AdEasier cells. Positive clones were confirmed by restriction digestion with *Pac*I, then transformed into Top 10F’ bacterial cells. The rAd-DEN2capsid_SPP_-prM/E-3ptmut virus was produced by transfection of 293T cells for production of high titers of recombinant adenoviruses encoding the DENV proteins (Earnest-Silveira et al., 2016a,b; Christiansen et al., 2019) as described above for the production of SARS-CoV-2 constructs.

One day prior to transfection, 293A cells (R70507, Thermo Scientific Aust Ltd) were seeded at 2.3 × 10^6^ cells per 10 cm^2^ dish, to reach confluency of approximately 80% next day. Transfection was then set up with Qiagen Effectene transfection kit (301427, Qiagen, Germany), with 4 μg of DNA per 10 cm dish. Following the protocol provided by the manufacturer, the plasmid DNA was added to 300 μL EC buffer and swirled gently. Thirty-two μL of Enhancer was then added to the reaction and incubated at room temperature for 2–5 min. Forty μL of Effectene transfection reagent was then added to the reaction and incubated for 5–10 min at room temp. This was followed by the addition of DMEM (Dulbecco’s Minimal Essential Medium, 10566024, Life Technologies), 10% fetal bovine serum (FBS) (12007C, Sigma, United States) and 60 μg/mL Penicillin (BenPenTM, CSL/Seqirus) and 100 μg/mL Streptomycin (Sigma, United States) up to 3 mL total. Media was removed from the cell culture dishes and cells were washed once with Phosphate Buffered Saline (PBS) very carefully. Seven mL of fresh media was then added to each dish. After incubation, the Effectene-DNA complex was added to each dish drop wise and dishes were incubated at 37°C, 5% CO_2_. Transfection efficiency and virus production was monitored by GFP expression daily.

By Day 7, fluorescence was observed in approximately 40% of cells and cells were starting to lift. Cells were dislodged and transferred to a 50 mL Falcon tube, spun down at 500 *g* for 5 min, supernatant removed and cells resuspended in 1 mL media per dish transfected. Four freeze–thaw–vortex cycles were performed to release adenovirus from cells. Samples were centrifuged at 3724 *g* at 4°C for 15 min to pellet the cell debris and the supernatant passed through a Whatman Filter Unit (0.45 μm, CLS431220, Sigma Aldrich, United States). The filtered viral supernatant (labelled as T1) was then used to infect 293A cells in 75 cm^2^ flasks at 0.5 mL of purified virus per flask. For infection, 0.5 mL of T1 passage virus was added to 3.5 mL media and a total of 4 mL was added dropwise to the cells. With constant and gentle rocking, infection was allowed to proceed for approximately 4 h, after which it was topped up to 12 mL with media. After approximately 4 days, cells started to lift and GFP expression was detected in approximately 60% of cells. Cells were dislodged and the process of intracellular virus collection was repeated, as described above. The supernatant was labelled as P1 (Passage 1) and stored at −80°C until used. The process of infecting and freeze-thawing was repeated up to Passage 3 or 4 in T175 cm^2^ flasks. As virus was passaged in 293A cells, the titre increased significantly with each passage, and passaging was stopped when the infection was ready in 1 day with 100% GFP observed and cells starting to lift.

### 2.2. Determination of rAd-SARS-CoV-2-SEM and rAd-DENcapsidSPP-prM/E-3ptmut viral titers

Vero (African green monkey kidney epithelial) cells were plated onto 12 well plates in duplicate at a density of 5 × 10^4^ cells per well in OptiPro Serum free media (1230901, Thermo Fisher, United States), 1% Pen/Strep, 2 × Glutamax (35050061, Thermo Fisher, United States) and were cultured overnight before infection the following day. Virus preparations were diluted in 300 μL OptiPro media, starting as 1 in 8 dilution with a two-fold serial dilution down to 1 in 8192. Plates were incubated for 4 h with gentle rocking to prevent cells from drying, and then topped up to 1.2 mL media. After 6 days, wells displaying 100% GFP expression without significant cell death were selected as the dilution of virus to be used for large scale infection. Titration of the virus was also set up following the TCID_50_ calculator. Vero cells were seeded at 1 × 10^4^ cells per well in 96 well plate. Virus was added in the dilutions 1 in 10 and diluted down 10-fold and FFU calculated by using the TCID_50_ calculator and by observing GFP.

### 2.3. Infection of Vero or Huh7 cells lines in serum-free media

Adapted Vero cells (2% FBS) or Huh7 cells were seeded into one 875cm^2^ 5-layer multi-flask (89204-478 VWR, United Kingdom) to reach 80% confluency the following day. Cells were washed twice with PBS and rAd-SARS-CoV-2-SEM or rAd-DENcapsid_SPP_-prM/E-3ptmut P3 virus was added to 20 mL of Optipro SFM, 1% Pen/Strep, 2 × Glutamax, and added carefully to the cells. After 4 h of incubation with gentle rocking, the flasks were then topped up to 100 mL total of Optipro media. The following day after infection, the cells were washed with at least 50 mL of PBS twice to wash off any Adenovirus and replaced with 100 mL of fresh Optipro Serum free medium. The infection was left for a further 2 days for DEN and 5 days for SARS-CoV-2 VLPs.

### 2.4. Purification of VLPs from Vero or Huh7 cells

Supernatant was collected from each multilayer flask and clarified at 3750 *g* for 10 min at 4°C. From this point all procedures were performed at 4°C or on ice. Supernatant was collected after clarification and centrifuged at 8000 *g* for 30 min at 4°C. The supernatant, containing VLPs, was then collected and subjected to sucrose cushion ultracentrifugation. To set up the sucrose gradient, 5 mL of sucrose solution (filtered 20% sucrose in PBS) was added to the bottom of UltraClear polypropylene tubes (344058, 38.5 mL, 25 × 89 mL, Beckman Coulter, United States) followed by 33 mL of supernatant. Tubes were balanced and then spun at 134000 *g* for 16 h at 4°C using a SW32Ti swinging rotor (Beckman Coulter, United States), with deceleration setting of 3. Immediately after spinning, the supernatant was discarded and 250 μL of sterile PBS pH 7.4 was added, tubes were left on ice and placed at 4°C with gentle shaking for 2 h to gently dissolve the pellet. The VLP samples were then transferred to Eppendorf tubes, quantified using the Bradford assay (Nanodrop 2000) and stored at −80°C in 20 μL aliquots. The VLPs were then checked and confirmed by Western Blot, ELISA and Electron Microscopy.

### 2.5. Fluorescence microscopy

Vero cells were maintained in DMEM (Thermo Fisher) supplemented with 2% FBS (GIBCO), 1 × GlutaMAX, (Thermo Fisher), 60 μg/mL Penicillin (BenPen^™^, CSL/Seqirus) and 100 μg/mL Streptomycin (Sigma). Cells were seeded into 4 × 24 well plates at a density of 5 × 10^4^ cells per well in duplicate for infection the following day. Virus dilutions of the adenovirus dengue construct or adenovirus SARS-CoV-2 constructs were added to the wells starting with 1 in 8 and diluting two-fold down. Virus was first diluted in 300 μL of media, added to the wells, and incubated for 4 h, after which they were topped up to 1.2 mL with media. GFP was observed over 3 days under a fluorescence microscope to detect MOI (Supplementary Figure 1).

### 2.6. Western immunoblot

DEN and SARS-CoV-2 VLPs harvested from culture supernatants were analysed by Western blot after separation by SDS-PAGE followed by transfer to PVDF membrane and probing with anti-spike (40591-T62, Sino Biologicals, China), anti-M (MBS434281, MyBioSource, United States) and anti-E (MBS8309656, MyBioSource, United States) antibodies for SARS-CoV-2 VLPs, and anti-E or anti-capsid antibodies for DEN VLPs as described previously (Earnest-Silveira et al., 2016a,b; Mukherjee et al., 2016). The mouse mAb antibody anti-E Flavivirus 4G2 (v150727-3284, Biotem, France) was supplied by Sanofi Pasteur, Aus/NZ. For DEN VLPs, the anti-E antibody was used at a dilution of 1 in 200 while the anti-capsid antibody (40263-T54, Sino Biologicals, China) was used at a dilution of 1 in 500. For SARS-CoV-2 VLPs, all antibodies were used at a dilution of 1 in 1000.

### 2.7. Electron microscopy

For negative staining and subsequent transmission electron microscopy (TEM) examination, 6 μL of VLP containing suspension or SARS-CoV-2 AUS/VIC01/2020 control material were applied directly to a glow-discharged 400-mesh copper formvar–carbon coated grid and allowed to adsorb for 20 s. The suspension was removed by blotting and then negatively stained using 3% phosphotungstic acid (pH 7.0). The negative-stained grids were blotted to remove excess stain and air-dried at room temperature. For DEN VLPs, a 10 μL sample was placed on an EM-copper grid with formvar/carbon coating for 5 min and washed once in a drop of PBS, and twice in a drop of deionised Milli-Q water. The grid was then placed on a drop of 1% of uranyl acetate for 5 min and gently dried with filter paper.

Before performing immuno-gold electron microscopy (IEM), the sample quality and concentration were verified using the above negative staining procedure. Copper 400-mesh glow-discharged formvar–carbon coated grids were placed onto a 10 μL drop of the VLP suspension or SARS-CoV-2 control and allowed to adsorb for 5 min before being washed three times in 10 mM HEPES/saline buffer (HN buffer). Washed grids were transferred to a 25 μL drop of HN buffer containing 1% bovine serum albumin (BSA) and incubated in a room temperature humidity chamber for 20 min. The grids were then blotted dry and immediately transferred to a 25 μL drop of HN buffer containing 0.2% BSA and polyclonal rabbit anti-SARS-CoV-2 RBD antibody (MBS2563840, My BioSource) diluted 1:500, then incubated for 1 h in a room temperature humidity chamber. After incubation the grids were washed three times in HN buffer containing 0.2% BSA and transferred to a 25 μL drop of HN buffer containing 0.2% BSA and monoclonal anti-rabbit antibody conjugated with 10 nm gold nanoparticles at a dilution of 1:20, then incubated for 1 h in a room temperature humidity chamber. Grids were washed five times in HN buffer containing 0.2% BSA followed by three rinses in 0.22 μm syringe filtered MilliQ H_2_O. The grids were then negative stained using 3% phosphotungstic acid (pH 7.0). The negative-stained grids were blotted to remove excess stain and air-dried at room temperature.

Negative stained grids were examined using an FEI Tecnai T12 Spirit electron microscope operating at an acceleration voltage of 80 kV. Electron micrographs were collected using an FEI Eagle 4 k CCD camera. File type conversion and morphometry were performed using the FEI TIA software package (Caly et al., 2020).

### 2.8. Atomic force microscopy

Atomic force microscopy images of SARS-CoV-2 and DEN VLPs were obtained as described previously (Collett et al., 2019), on a Cypher ES AFM (Oxford Instruments, Asylum Research, Santa Barbara, CA, United States) using small amplitude (AM)-AFM. Samples were diluted to between 1 and 10 μg/mL in Tris-buffered saline (TBS, Sigma, >99%) to ensure imaging of single particles. After being filtered through 0.45 μm filters (Millipore), samples were transferred to a freshly cleaved muscovite (mica) surface in a droplet of approximately 100 μL of TBS. Biolever BL-AC40TS cantilevers (Oxford Instruments, Asylum Research, Santa Barbara, CA, United States, nominal spring constant kc = 0.09 N/m) were used, calibrated prior to each experiment via the thermal spectrum method, and the lever sensitivity was determined using force spectroscopy. The spring constant (kc) was determined for each cantilever used, with values in the range of 0.05–0.1 N/m obtained for all cantilevers. Elasticity of DEN VLPs was determined by Force Spectroscopy, as described previously (Collett et al., 2019, 2021), by fitting force versus distance curves (FD curves) obtained from the central region DEN VLPs.

### 2.9. ELISA assay for determination of reactivity of SARS-CoV-2 VLPs

Purified VLPs were tested by coating 96-well, flexible, flat-bottomed PVC microtiter plates (442,404, Nunc, United States) with 50 μL of VLP ranging from 0.31 to 10 μg/mL in carbonate coating buffer (100 mM Na_2_CO_3_ and NaHCO_3_, pH 9.6) and incubated overnight at 4°C. Coating solution was then discarded and wells were blocked with blocking buffer (2% BSA in PBS) and incubated for 1 h at 37°C. Plates were then washed 4 times with PBS supplemented with Tween of 0.05% and blotted dry. Primary antibody (Rabbit anti-SARS-CoV-2) Spike RBD Polyclonal Antibody (MBS2563840, My BioSource) was made in two-fold serial dilutions in blocking buffer from 1 in 800 down to 1 in 102400. Fifty microlitres of the antibody was added to the appropriate wells and plates incubated for 1 h at 37°C. Plates were then washed again four times and 50 μL of goat anti- rabbit secondary antibody conjugated to horseradish peroxidase (HRP) (P0448, Dako) at 1/2500 was made up in blocking buffer and added to each well. After incubation for 1 h at room temperature, plates were washed again with PBST 0.05% and blotted dry. Fifty microlitres of tetramethylbenzidine (TMB) substrate (002023, Thermofisher Scientific) was added to each well and incubated for 10 to 15 min at room temperature before stopping the reaction by adding 50 μL/well of 0.16 M H_2_SO_4_. Absorbance values were determined on a Labsystems Multiskan Multisoft plate reader (Thermo Scientific) at 450 nm.

### 2.10. ELISA assay for determination of reactivity of DENV VLPs

Reactivity of purified DENV VLPs were confirmed by ELISA using DENV HuMAbs, C10 and A11 (Dejnirattisai et al., 2015). Fifty microlitres of VLP per well at a final concentration of 5 μg/mL was added to 96-well, flexible, flat-bottomed PVC microtiter plates (442,404, Nunc, United States) in carbonate coating buffer. Plates were incubated at 4°C overnight in a humidified chamber. On the second day, the coating solution was discarded, and plates blocked with 1% BSA (A2153, Sigma, United States) in PBS for at least 2 h at room temperature. The plates were then washed 4 times with PBS and blotted dry. DENV HuMAbs, C10 and A11 were diluted in PBS with 0.5% BSA/PBS at 100 μg/mL, and 50 μL of each antibody was added to wells and incubated for 2 h at room temperature. Plates were washed 4 times with PBS and blotted dry. Fifty microlitres of anti-human antibody conjugated to HRP (62-8420, Invitrogen, United States) at 2 μg/mL in 0.5% BSA/PBS was added to each well. The plates were incubated for 1 h at room temperature then washed 4 times with PBS. Fifty microlitres of tetramethylbenzidine (TMB) substrate (002023, Thermofisher Scientific) was added to each well and incubated for 10–15 min at room temperature before stopping the reaction by adding 50 μL/well of 0.16 M H_2_SO_4_. Absorbance values were determined on a Labsystems Multiskan Multisoft plate reader (Thermo Scientific) at 450 nm.

### 2.11. Self-adjuvanted SARS-CoV-2 and DEN VLP production

Vero cells grown to 80% confluency in DMEM (Thermo Fisher) supplemented with 2% fetal calf serum (FCS, GIBCO) were infected with rAd-SARS-CoV-2-SEM or rAd-DEN3capsidSPP-prM/E-3ptmut virus at an MOI of 1.0 with or without α-GalCer (KRN7000 - Enzo Life Sciences) at 0, 10, 100 or 1000 ng/mL. Cell culture supernatants were collected after 3 days for DENV and 6 days for SARS-CoV-2 and VLPs isolated as described above.

### 2.12. Mice

C57BL/6 mice were bred in-house at the Peter Doherty Institute for Infection and Immunity, Biological Research Facility, Melbourne, Australia. All mice used were 6–10 weeks old, male mice were used for SARS-CoV-2 immunisations, female mice were used for all other experiments. All procedures were approved by the University of Melbourne Animal Ethics Committee.

### 2.13. *In vitro* VLP experiments

To investigate NKT cell activation by VLP vaccine formulations, VLPs were cultured at 37^o^ C, 5% CO_2_ overnight with splenocytes from naïve C57BL/6 mice, at 3 × 10^6^ cells per well in 48-well flat-bottom plates (500 μL). The next day, cells were harvested and washed with fresh media (800 μL), then re-seeded in 96-well round-bottom plates (150 μL) at 3 × 10^5^ per well and cultured for an additional 72 h without VLPs prior to FACS analysis.

### 2.14. *In vivo* experiments

For DEN and HCV VLP immunisations, mice were immunised with two doses of 10 μg DEN-3 VLP (delivered with Alum), α-GalCer-DEN-3 VLP, HCV VLP (delivered with Alum), or α-GalCer-HCV VLP intramuscularly or subcutaneously 2 weeks apart. Mice were bled from the tail vein immediately before the booster dose. One week after the booster, blood and spleens were harvested for analysis.

For SARS-CoV-2 immunisations, mice were immunised intramuscularly with two doses of 20 μg of either SARS-CoV-2 VLP, α-GalCer-SARS-CoV-2 VLP, or SARS-CoV-2 VLP delivered with AddaVax (vac-adx-10, Invivogen, United States) or PBS or α-GalCer alone as controls. Mice were either (Carreno et al., 2014) immunised at days 0 and 7, bled at day 0 and harvested on day 10; (Mascola and Fauci, 2020) immunised at day 0 and day 14, bled at days 0 and 14 and harvested on day 21; or (Rauch et al., 2018) immunised at day 0 and day 14, bled at day 0, 14, 28 and 56, and harvested on day 70.

Serum was used for the evaluation of antibodies by ELISA, using either DEN or SARS-CoV-2 VLP, or RBD peptide as coating antigen. Splenocytes were collected for the analysis of the T cell and B cell responses in mice immunised with VLPs by flow cytometry and ELISpot assay, respectively.

Processing of spleens was as follows: Spleens were placed into Petri dishes with 0.5% PBS/BSA and macerated with the plunger of a 5 cc syringe. This was then transferred to a 70 μm strainer placed over a 50 mL tube. Media RF10 [RPMI 1640 medium (61870127, Thermofisher Scientific), 10% fetal calf serum (10099141, qualified, Australia), 7.5 mM HEPES, 76 μM 2-mercaptoethanol (M6250, Sigma, United States), 150 U/mL penicillin, 150 mg/mL streptomycin (Sigma), 150 μM non-essential amino acids (11140050, Thermofisher Scientific)] was added a few mL at a time, up to 12 mL while using the plunger of syringe to squeeze/macerate the spleen through the strainer. The suspension was centrifuged at 500 g on a desktop centrifuge at 4°C for 7 min and decanted. Four mL of Hybri-max Lysis Buffer (R7757, Sigma, United States) was added and incubated for 1 min at RT with gentle mixing. This was spun down again at 500 g for 7 min at 4°C and decanted. The cells were resuspended gently in cold PBS and spun down at 500 g for another 7 min and then resuspended in 15 mL RPMI and counted using the trypan blue method. For ELISpot assay (described below), cells were then transferred to 4 wells of a 12 well plate at 1 mL/well, 5 × 10^6^ cells/well and topped up with RF10 [supplemented with 10 ng/mL interleukin 2 (IL-2)]. Twenty micrograms of DEN VLP or SARS CoV2 VLP was then added to 2 wells for stimulation and the plate was incubated for 5 days in a 37°C humidified incubator with 5% CO^2^.

### 2.15. B cell ELISpot assay

The ELISpot assay was performed with a Mouse IgG Basic Kit (3825-2H, Mabtech, United States) to determine the number of DEN VLP specific B cells from spleen cells of immunised mice. The ELISpot plate (MSIPS4510, Millipore, United States) was prepared by pre-wetting with 35 μL 35% ethanol v/v in water per well for a maximum of 2 min. The plate was then washed 5 times with sterile water (200 μL/well). 100 μL of DEN VLP (1 μg) or SARS-CoV-2 VLP (1 μg) was added to each well and incubated overnight at 4°C. The plates were rinsed 5 times with sterile PBS to remove excess antigen. Plates were blocked with 200 μL/well of RF10 medium for at least 30 min to 2 h at room temperature. For analysis of *in vivo* activated cells, media and stimulated cells were removed from the 12 well plate (described above, section 2.14). Splenocytes from each mouse were transferred into a 15 mL tube, washed twice with PBS, pelleted, and then resuspended gently in 1 mL of RP10 containing 10 ng/mL interleukin 2 (IL-2). The cells were then gently pelleted and resuspended in media at the desired cell number required for the ELISpot wells. Media was removed from the ELISpot plate and cells added to the wells containing RP10 supplemented with IL-2. Plates were incubated in a 37°C humidified incubator with 5% carbon dioxide for 24 h. The next day, media was removed, and plates washed 5 times with PBS (200 μL/well). The cells were then incubated for 2 h at room temperature with secondary antibody (1 μg/mL anti-IgG-biotin antibody) in 0.5% FCS/PBS. The plate was washed 5 times with PBS and cells then incubated with 100 μL/well of streptavidin-HRP in 0.5% FCS/PBS for 1 h at room temperature. The plate was washed 5 times with PBS and 100 μL/well of TMB substrate solution was added, and developed until spots emerged (20–30 min). Colour development was stopped by washing with tap water.

### 2.16. Interferon-γ ELISpot assay

Analysis of Interferon-γ (IFN-γ) -secreting cells was carried out using the IFN-γ ELISpotPlus (HRP) kit (3321-4HPT-2, Mabtech, United States) with plates supplied precoated with anti-IFN-γ capture monoclonal antibody (AN18). After washing wells with 200 μls sterile PBS per well, plates were then blocked with RF 10 medium for at least 30 min. Splenocytes stimulated for 5 days with 20 μg total VLPs were harvested, washed and added to the wells at 5 × 10^5^ cells/well. Forty-eight hour later, plates were washed with PBS and biotinylated anti-IFN-γ capture antibody (mAb R4-6A2) added at a final of 1 μg/mL and incubated for 2 h at room temperature. Plates were then washed and streptavidin conjugated horse-radish peroxidase (Mabtech, United States) at 1:1000 was added and incubated for 1 h. Spots representative of IFN-γ-producing cells were developed by the addition of TMB and washing with water after development.

### 2.17. ELISA assay for determination of VLP-specific and RBD-specific antibody responses from immunisations with SARS-CoV-2 VLPs

ELISA plates were coated with 50 μL/well of VLP at 20 μg/ml or RBD (Deliyannis et al., 2022) at 5 μg/mL in PBS and incubated at 4°C overnight in a humidified chamber. The coating antigens (VLP and RBD) was then discarded and wells were blocked with 100 μL of 2% BSA/PBS for 1 h at 37°C in a humidified chamber. Immunised mice serum was initially diluted 1 in 10 in blocking buffer and serially diluted. Fifty microlitres of each serum dilution was added to coated wells. Plates were incubated for 1 h at room temperature and wells were then washed four times with PBS with 0.05%Tween20. Fifty microlitres of anti-mouse antibody conjugated to HRP (AB97046, Abcam, United States) diluted 1 in 10,000 was then added to wells and incubated at room temperature for 1 h. Plates were then washed 4 times with PBS with 0.05%Tween20, and developed by adding 50 μL of Tetramethylbenzidine (TMB) substrate (002023, Thermofisher Scientific) and stop solution as described above. Absorbance values were determined on a Labsystems Multiskan Multisoft plate reader (Thermo Scientific) at 450 nm.

### 2.18. ELISA assay for determination of VLP-specific and antibody responses from immunisations with DENV and HCV VLPs

ELISA plates were coated with 50 μL/well of VLP at 5 mg/mL in carbonate coating buffer and incubated at 4°C overnight in a humidified chamber. The coating antigen (VLP) was then discarded and wells were blocked with 100 μL of 1% BSA/PBS for 1 h at 37°C in a humidified chamber. Immunised mice serum was initially diluted 1 in 10 in blocking buffer and serially diluted. Fifty microlitres of each serum dilution was added to coated wells. Plates were incubated for 1 h at room temperature and wells were then washed four times with PBS with 0.05%Tween20. Fifty microlitres of anti-mouse antibody conjugated to HRP (P0161, Dako) diluted 1 in 400 was then added to wells and incubated at room temperature for 1 h. Plates were then washed 4 times with PBS with 0.05%Tween20, and developed by adding tetramethylbenzidine (TMB) substrate (002023, Thermofisher Scientific) and stop solution as described above. Absorbance values were determined on a Labsystems Multiskan Multisoft plate reader (Thermo Scientific) at 450 nm.

### 2.19. Flow cytometry

The phenotype of immune cells was assessed following 30 min incubation at 4°C with antibody-cocktails containing combinations of α-GalCer (C24:1)/(PBS-44)-loaded CD1d-tetramer [PE (BD Biosciences) or BV421 (BioLegend)], 7-aminoactinomycin D [7-AAD (Sigma-Aldrich)], anti-CD16-CD32 (2.4G2, grown in-house), monoclonal antibodies (mAb) against CD8α-BUV805 CD19-BV786, CD44-FITC, CD62L-APC and TCRβ-BV711 (BD Biosciences) or TCRβ-A647 (BioLegend) (Supplementary Figure 4). Cells were then washed twice with PBS + 2% v/v FBS prior to flow cytometry using an LSRFortessa analyser (BD Biosciences). Data was analysed using FlowJo software (Tree Star).

### 2.20. Data processing and statistical analysis

Processing of AFM data involved using a combination of the Asylum Research software, custom MATLAB codes, and the Gwyddion software package (Nečas and Klapetek, 2012). Height and diameter values were analysed after filtering data with arbitrary cut-off values of >10 nm and > 20 nm, respectively, to exclude single proteins and other debris but include all intact and partially intact VLPs. Further data processing and statistical analysis (ANOVA and Mann–Whitney tests) was performed using GraphPad Prism.

## 3. Results

### 3.1. Assembly of SARS-CoV-2 VLPs

**Figure 1:**
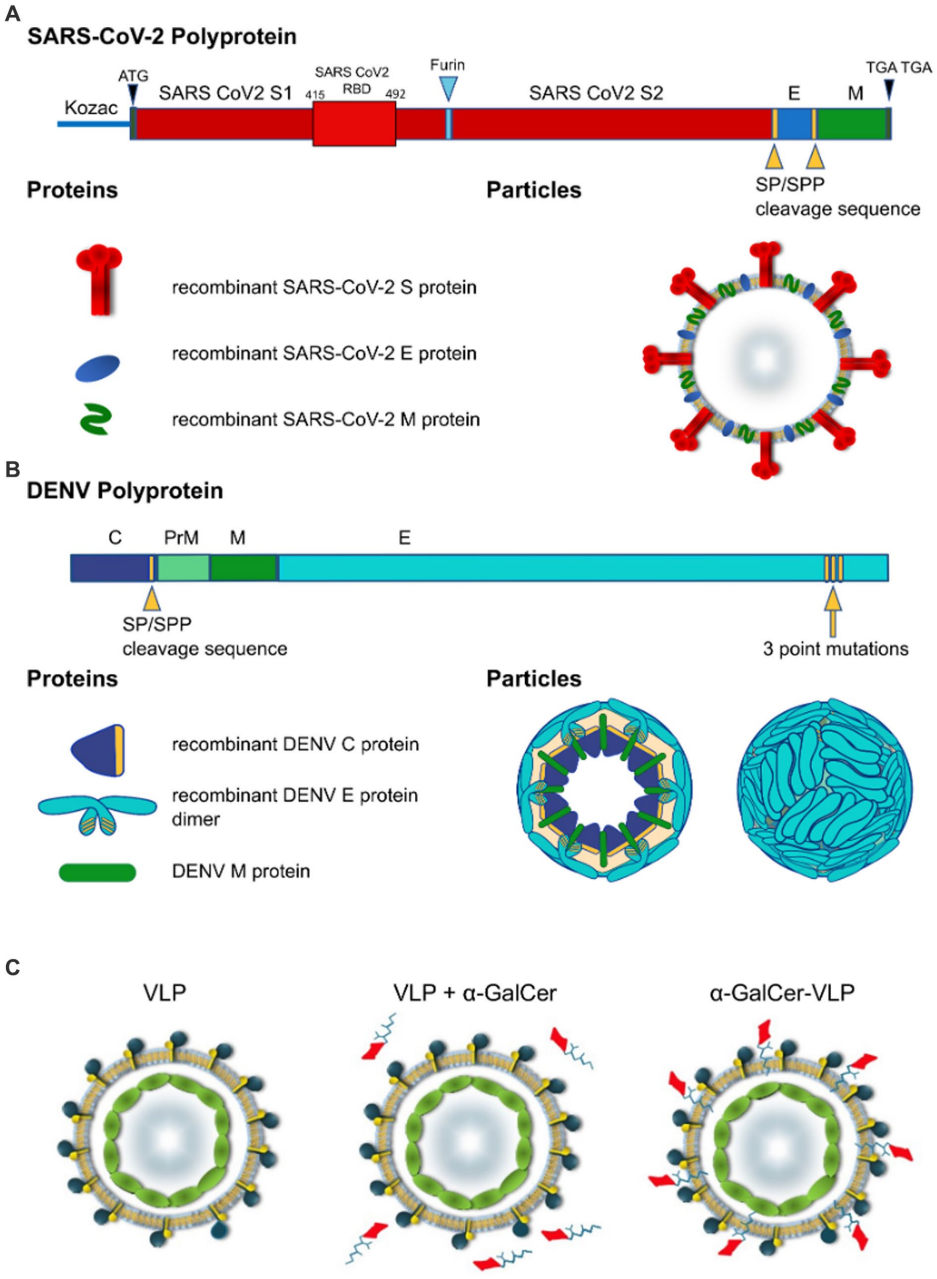
Design of self-cleaving and self-adjuvanting VLPs. Polyprotein and particle organisation of **(A)** SARS-CoV-2 VLP, and **(B)** DEN VLPs. Yellow arrowheads indicate signal peptidase/signal peptide peptidase (SP/SPP) cleavage sequence, blue arrowhead indicates furin cleavage sequence, Yellow arrow indicates 3 point mutations introduced to the C terminus of the DENV polyprotein. Modifications to the polypeptide/protein sequence are indicated in yellow, beige in DEN particle cross-section indicates lipid membrane. SARS-CoV-2-S, spike protein; E, envelope protein; M, membrane protein; C, capsid protein (DENV); prM, precursor membrane protein (DENV). **(C)** VLP vaccines can be delivered alone or mixed with adjuvants and agonists like α-GalCer added to purified VLPs. In contrast to these approaches, we have incorporated α-GalCer directly into the particle during intracellular VLP assembly to produce unique self-adjuvanted VLP vaccines.

**Figure 2:**
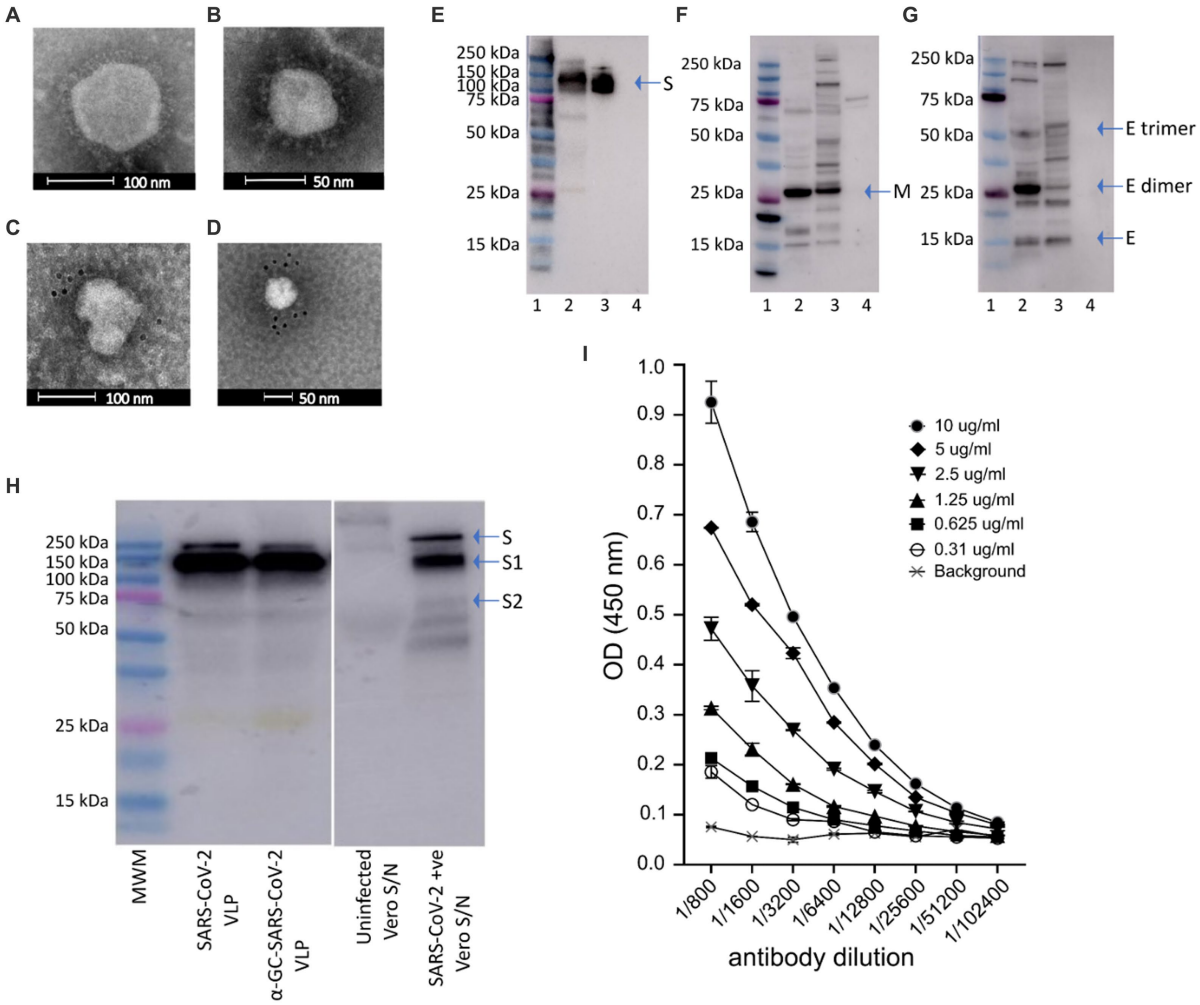
Electron micrographs, Western blot analysis and ELISA of purified of SARS-CoV-2 VLPs. **(A,B)** Electron micrographs showing SARS-CoV-2 VLP sizes ranging approximately 50–110 nm. Immunogold with gold nanoparticle labelled anti-RBD monoclonal antibody confirming **(C)** the presence of spike protein on SARS-CoV-2 VLPs, and **(D)** SARS-CoV-2 virus as positive control. **(E–G)** Western blot analysis of purified SARS-CoV-2 VLPs. Blots were probed with polyclonal anti-S **(E)**, anti-M **(F)** or anti-E **(G)** antibodies. Bands representing the monomeric, dimeric and trimeric forms of the E protein can be seen in **(G)**. In each blot lane 1 = molecular weight marker (MWM), lane 2 = SARS-CoV-2 VLP, lane 3 = positive control, lane 4 = negative control. **(H)** Western blot analysis of SARS-CoV-2 VLPs, SARS-CoV-2 VLPS with α-GalCer incorporated, supernatant from uninfected vero cells (as negative control) and vero cells infected with SARS-CoV-2 virus (as positive control). Blots were probed with polyclonal anti-S antibody. **(I)** Concentrations of SARS-CoV-2 VLPs ranging from 0.3 to 10 μg/mL were probed with a serial dilution of anti-RBD.

Proper processing of the translated polypeptide during natural infection with both DENV and SARS-CoV-2 involves cleavage of the viral proteins by viral and cellular proteases. To overcome the requirement for cleavage by viral proteases, cleavage sequences were replaced by a sequence derived from HCV that is cleaved by an endoplasmic reticulum (ER) signal peptide peptidase (SPP) (Oehler et al., 2012). Introduction of the HCV core protein cell signalase cleavage sequence allows for the efficient release and self-assembly of virus structural proteins. For SARS-CoV-2 VLPs we produced a gene construct containing the full-length S separated from the E gene by the SP/SPP sequence (Oehler et al., 2012). The same SP/SPP sequence was also introduced between the E and M genes (Figure 1A). The resultant polyprotein undergoes cleavage in the endoplasmic reticulum, releasing separate S, E and M proteins that self-assemble into VLPs for secretion into culture supernatant as shown by electron microscopy (EM) of SARS-CoV-2 VLPs purified from culture supernatant (Figures 2A,B). The SARS-CoV-2 VLPs ranged in size from approximately 50–110 nm and were surrounded by characteristic spikes. Furthermore, immunogold EM showed the binding of gold beads to the tips of spikes on the SARS-CoV-2 VLPs with anti-RBD mAb (Figure 2C), similar to SARS-CoV-2 virus (Figure 2D).

Western blot analysis of purified SARS-CoV-2 VLPs probing with anti-S, anti-E and anti-M antibodies showed the presence of Spike (140–180 kDa), Envelope (8.4 kDa) and Membrane (25 kDa) proteins in purified SARS-CoV-2 VLPs (Figures 2E–G). Furthermore, probing with polyclonal anti-S antibody showed the presence of S1 protein analogous to S1 present in SARS-CoV-2 virus and the incorporation of α-GalCer into the VLPs did not affect the size of S1 (Figure 2H).

To further characterise the SARS-CoV-2 VLPs, we tested purified VLPs by ELISA (Figure 2I). Plates were coated with increasing concentrations of SARS-CoV-2 VLPs ranging from 0.3 μg/mL to 10 μg/mL. Plates were then probed with a serial dilution of anti-RBD mAb. SARS-CoV-2 VLPs were strongly reactive with the anti-RBD Mab and remained reactive down to the lowest coating concentration of 0.3 μg/mL (Figure 2I).

### 3.2. Assembly of DEN VLPs

DEN VLPs contained C/prM/E DENV proteins from serotypes 2, 3 and 4. First, we developed the rAd-DENcapsid-prM/E of serotype 2 (16681 clone; DENV-2 VLP) construct to optimise production techniques before producing VLPs for other serotypes. The SP/SPP sequence (Oehler et al., 2012) was again introduced to ensure that the cleavage of the DEN capsid protein was driven by the host cell SP/SPP, thereby removing the requirement for the inclusion of the DENV-2 NS3 protease. Consequently, the capsid protein was released to allow for the self-assembly of C/M/E VLPs (Figure 1B).

**Figure 3:**
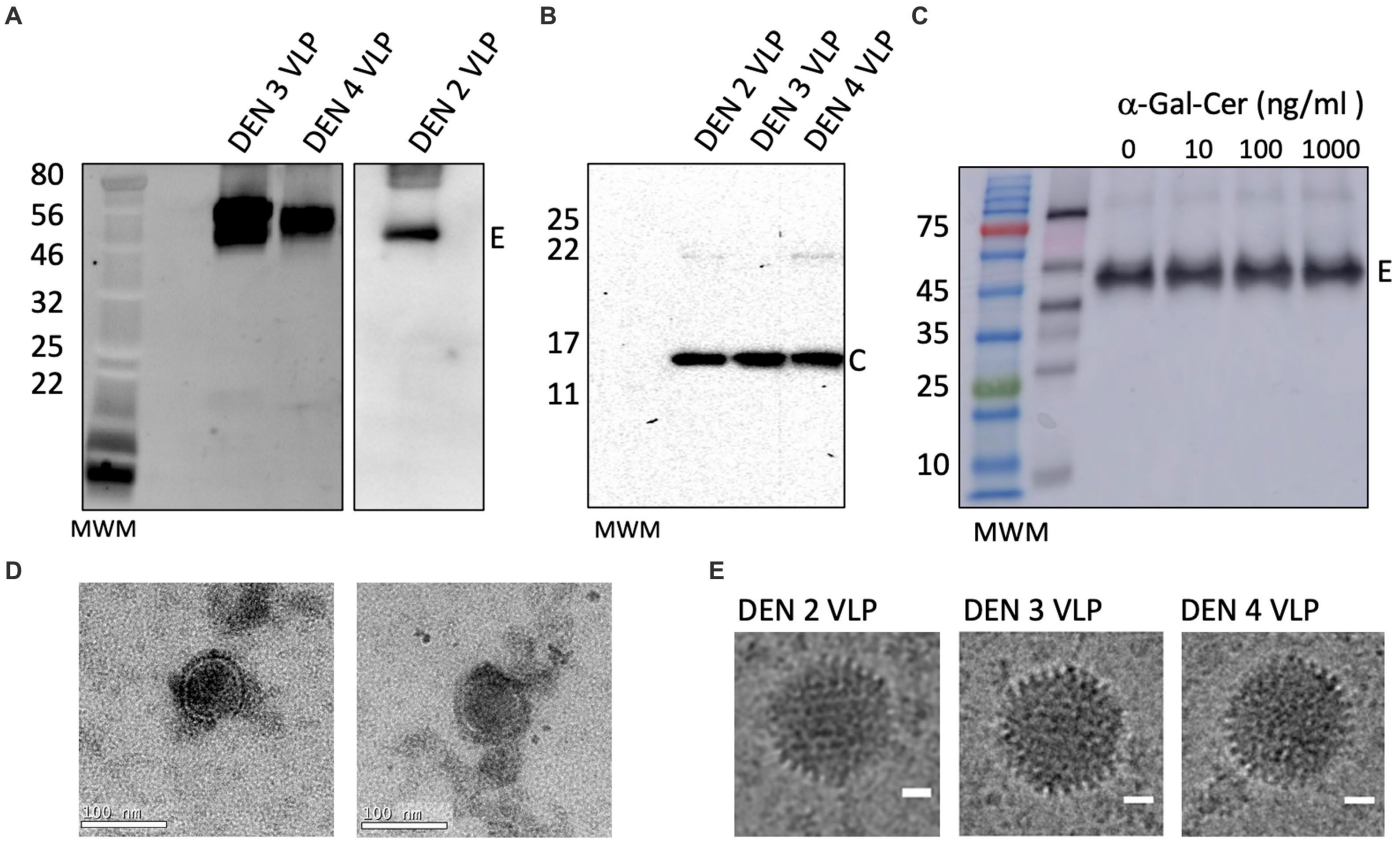
Verification of large-scale production of DEN VLPs. Western blot of DEN VLPs from a large-scale preparation of VLPs purified from cell culture supernatants, showing **(A)** envelope and **(B)** capsid proteins. Molecular weight marker (MWM) is shown. **(C)** Western blot of α-GalCer-DEN-4 VLP purified from cell culture supernatants by ultra-centrifugation and probed with anti-E antibody (1/500). **(D)** Transmission electron micrographs of purified DEN-2 VLPs. Scale bar (bottom right of image) represents 100 nm. **(E)** Cryogenic electron micrographs of DEN-2, DEN-3, and DEN-4 VLPs. Scale bar represents 20 nm.

To improve the release of the DEN E protein from the ER, and thereby prevent the VLPs from being retained intracellularly, the DEN E protein ER retention signal was altered by introducing three point mutations in the E stem anchor (coding for I398L, M401A, and M412L substitutions) that have previously been shown to enhance the release of the DEN E protein from the ER (Purdy and Chang, 2005; Hsieh et al., 2008) (clone rAd-DEN-2capsid_SPP_-prM/E-3ptmut). This modification allowed for the efficient production and release of the DEN E protein from the ER in transduced mammalian cells and the subsequent self-assembly and secretion of DEN VLPs (Figure 3).

We next produced rAd-DENcapsid_SPP_-prM/E-3ptmut viruses for serotype 3 and 4 using the cloning and amplification strategies described above. DEN-3 and DEN-4 VLPs were examined by Western blot to confirm the presence of Envelope (55 kDa) and Capsid (13 kDa) proteins in purified VLPs (Figures 3A,B). Also, the addition of increasing concentrations of α-GalCer did not alter the size or migration of E protein (Figure 3C). Transmission electron micrographs of DEN-2 VLPs (Figure 3D) and cryo-electron micrographs of DEN-2, DEN-3 and DEN-4 VLPs (Figure 3E) showed particles of the expected size and morphology, indicating that self-assembly of DEN-2 proteins led to VLPs displaying morphology consistent with native virus.

### 3.3. Infection of high furin expressing cells leads to optimal DEN VLPs maturation

For the assembly of DENV particles, cleavage of the prM protein within the trans-Golgi by host-derived furin protease is required. To see if the production of the DEN-2 VLPs could be facilitated, we produced a stable human furin expressing Vero cell line (Vero-Furin), as this has recently been shown to enhance the production of mature dengue virus (Mukherjee et al., 2016). To determine the optimal cell line for the production of DEN VLPs we infected Vero cells (a low furin expressing cell line), Vero-Furin (Mukherjee et al., 2016) and Huh7 cells (high furin expressing cell lines) (Tay et al., 2012) with rAd-DEN2coreSPP-prM/E-3ptmut virus. Infection of Vero, Vero-Furin and Huh7 cells with virus all resulted in high transduction levels, as indicated by the high proportion of cells expressing green fluorescent protein (GFP) (Supplementary Figure 1).

### 3.4. DEN VLPs present conformational epitopes on envelope protein domains 1 and 2

To gain further insight into whether conformational neutralising epitopes of the envelope protein that are important for DENV neutralisation are presented on the VLPs similar to native virus, we used mAbs directed at the DENV envelope protein (Dejnirattisai et al., 2015) and tested the binding of these HuMAbs to DEN-2 VLPs by ELISA using methods as previously described (Christiansen et al., 2018a,b, 2019). The first HuMAb, C10, is known to bind to neutralising epitopes in the envelope dimer epitope 1 (EDE1), whilst the second HuMAb, A11, binds to neutralising epitopes in the envelope dimer epitope 2 (EDE2) domain of the viral envelope (Dejnirattisai et al., 2015).

**Figure 4:**
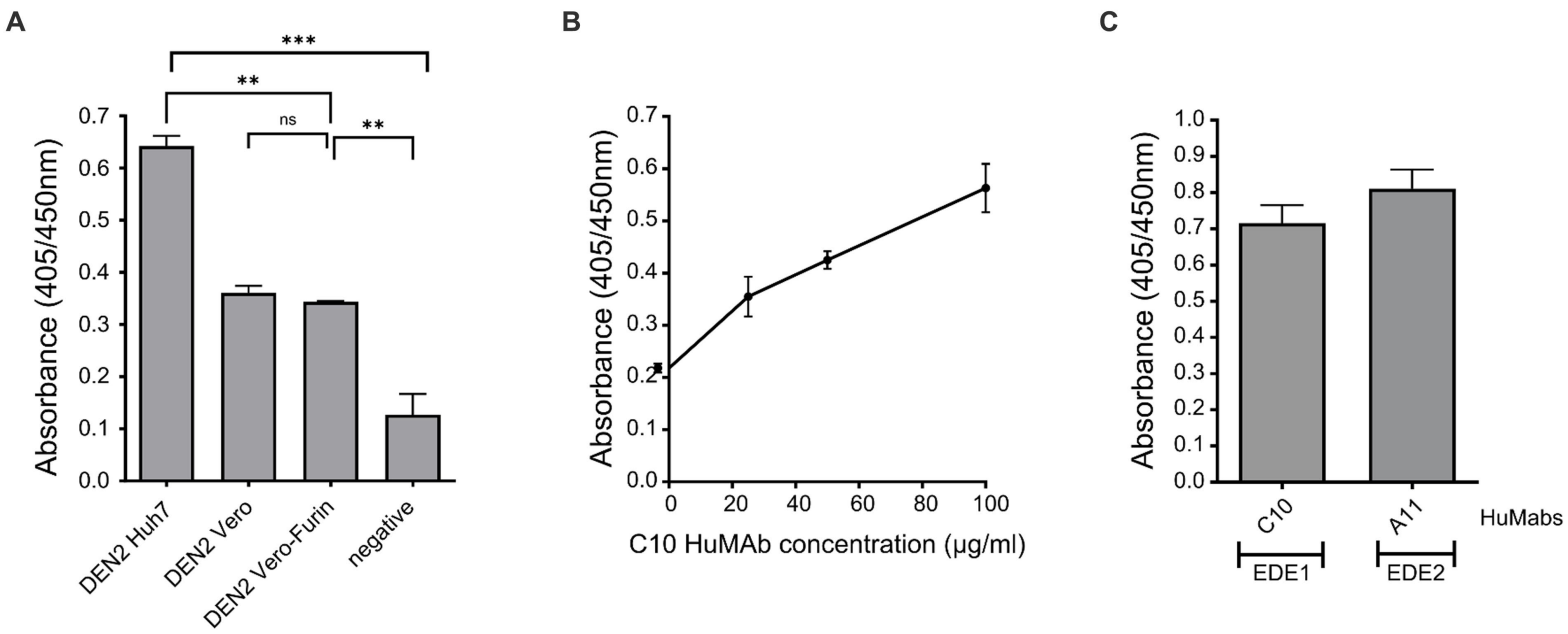
Assessment of the effect of host cell derivation and monoclonal antibody binding to DEN VLPs, as determined by ELISA assay. **(A)** Binding of HuMAb C10 to DEN-2 VLPs produced in Huh7 cells, Vero cells and Vero-Furin cells (negative control PBS). **(B)** Titration of C10 binding to DEN-2 VLPs. **(C)** Binding of C10 and A11 to DEN-2 VLPs. One-way ANOVA was used to determine significance in **(A)** (*p* < 0.05, * < 0.05, ** < 0.005, *** < 0.0005, **** < 0.0001).

We first tested the binding of C10 to DEN-2 VLPs produced in Huh 7, Vero and Vero-Furin cells (Figure 4). The strongest binding was observed to DEN-2 VLPs produced in Huh 7 cells, and was significantly higher than that observed to VLPs produced in both Vero and Vero-Furin cells (Figure 4A). Binding of HuMAb C10 to DEN-2 VLPs increased in a dose-dependent manner (Figure 4B). This is important as it shows our VLPs present conformationally dependent epitopes in a manner recognised by broadly neutralising HuMAbs, inferring the potential of these VLP vaccines to include broadly protective Ab responses. The binding of the EDE2 HuMAb, A11, was not significantly different to the binding of the EDE1 HuMAb, C10 (Figure 4C). Based on these results, DEN VLPs produced in Huh7 cells were used for further characterisation. The recognition of surface epitopes displayed on our DEN VLPs by HuMAbs reaffirms the correct assembly of these particles, which is vital for eliciting NAb responses.

### 3.5. Detailed biophysical characterisation of SARS-CoV-2 and DEN VLPs by AFM

**Figure 5:**
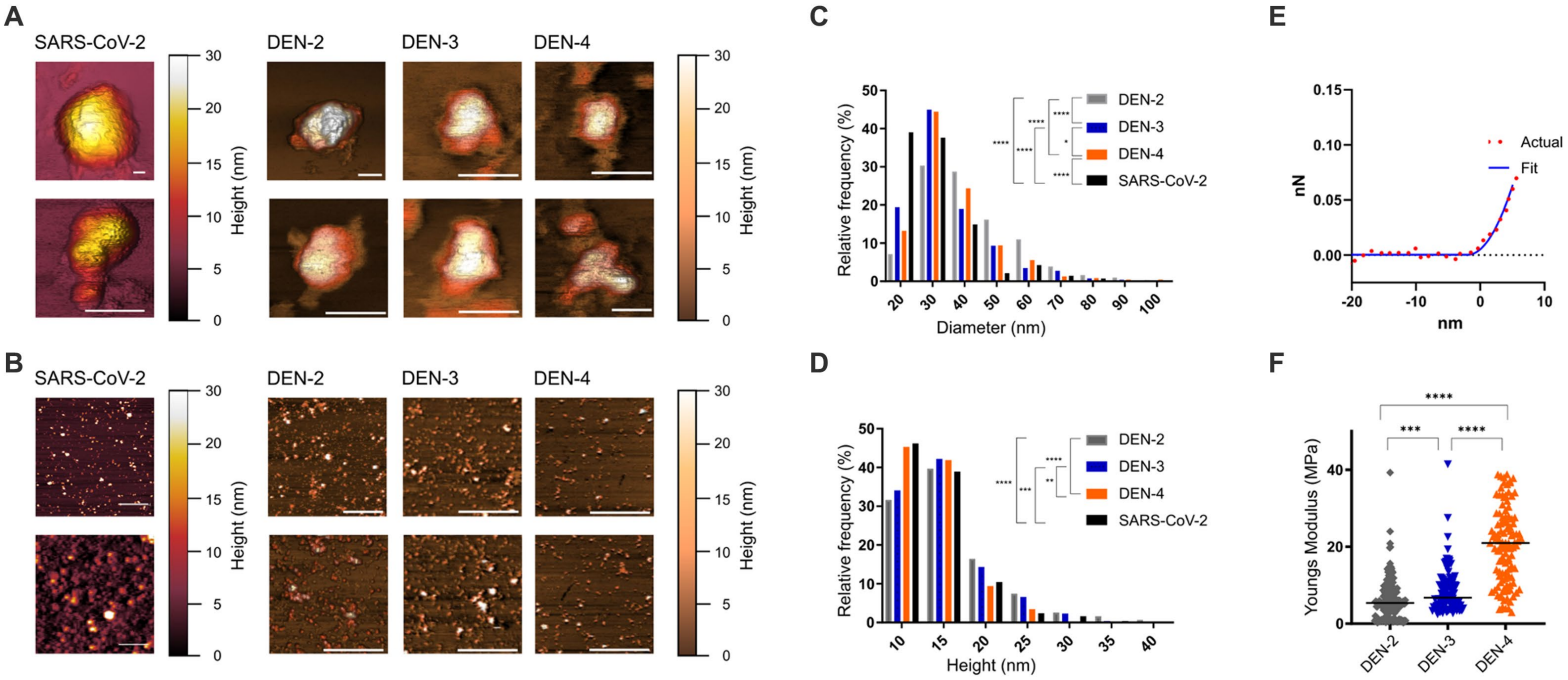
Nanoscale imaging and biophysical characterisation of SARS-CoV-2 and DEN VLPs by AFM. **(A)** High resolution AFM images (100–500 nm scan sizes) show ultrastructural detail of individual VLPs. White scale bars (bottom right of each image) represent 50 nm, false colour scale bar represents 30 nm *Z* axis (height). Scans of individual particles show particle diameter of between 50 and 300 nm. **(B)** AFM images at larger scan sizes (1–2 μm) show overall morphology and size of VLPs. White scale bars (bottom right of each image) represent 500 nm, false colour scale bar represents 30 nm *Z* axis (height). Height and diameter statistics were calculated from these and similar images. Distribution of diameter **(C)** and height **(D)** of SARS-CoV-2 and DEN VLPs for each serotype produced. **(E)** Representative force/distance curve collected from DEN-2 VLP showing distance as a function of force during nanoindentation of the VLP. Actual data (red circles) compared to data fitted to the Sneddon/Hertz equation (blue line), used to calculate Young’s elastic modulus. **(F)** Distribution of calculated Young’s elastic modulus for each of the DEN VLP serotypes. Black line indicates the mean on each scatter plot. Statistically significant differences between serotypes were determined using the Mann–Whitney test and are indicated by asterisks (*p* < 0.05, * < 0.05, ** < 0.005, *** < 0.0005, **** < 0.0001).

AFM provides a powerful tool for visualising surface topographies of the VLPs and allows for accurate determination of particle sizes. Nanoscale images were collected from SARS-CoV-2 VLPs and the three serotypes of DEN VLPs. Topographical AFM scans were obtained at 100–300 nm scan sizes to capture ultrastructural detail of individual VLPs or small clusters of VLPs (Figure 5A) and at 1 and 2 μm scan sizes for statistical analysis of particle sizes (Figure 5B and Table 1).

**Table 1:** Mean height and diameter, with standard deviation, for all VLP serotypes.

Virus/serotype	Height		Diameter	
	Mean (nm)	Std. dev.	Mean (nm)	Std. dev.
DEN-2 (*n* = 310)	16.0	5.5	41.9	13.8
DEN-3 (*n* = 258)	15.1	4.6	34.7	12.0
DEN-4 (*n* = 218)	13.9	3.4	36.4	12.6
SARS-CoV-2 (*n* = 249)	14.0	4.17	27.7	5.9

When measured by AFM, SARS-CoV-2 VLPs were found to have a mean height of 14 nm and a mean diameter of 27.7 nm. For DEN VLPs, mean particle heights ranged from 13.9 (DEN-4) to 16.0 nm (DEN-2) across the serotypes, while mean diameters were found to fall between 34.7 (DEN-3) and 41.9 nm (DEN-2) (Table 1). An ANOVA showed significant differences in height and diameter between DEN VLPs of all serotypes. However, as data did not follow a normal distribution, a non-parametric Kruskal-Wallis test was performed and similarly showed significant differences in height and diameter between DEN VLPs of all serotypes. Pairwise comparisons were therefore performed using the Mann–Whitney test (correcting for multiple comparisons). A significant difference (*p* < 0.05) in height was found between DEN-2 and DEN-4 VLPs, and between DEN-3 and DEN-4 VLPs, but not between DEN-2 and DEN-3. A significant difference in height was found between SARS-CoV-2 VLPs and DEN-2 and DEN-3, but not DEN-4 VLPs. A significant difference in diameter (*p* < 0.0001) was found between all DEN VLPs, and between SARS-CoV-2 VLPs and all DEN VLP serotypes. However, while DEN-2 VLPs have the largest mean height and diameter of the genotypes investigated (Table 1), the overall shape of size distribution was similar for all serotypes (Figures 5C,D). Scans of individual particles show particle diameters of between 50 and 300 nm (Figure 5A). These values are higher than the statistical averages of the AFM data and the values obtained by TEM. The discrepancy is due to the capability of AFM to collect accurate size information from the entire range of particles and particle fragments within a sample, potentially including aggregates of VLPs, while TEM images of clearly defined individual particles were used to derive size. However, overall agreement is seen in images of individual particles with diameters between 50 and 100 nm.

AFM can also be used as a nanoindentation tool to measure the elasticity of a material, which provides an indication of particle stability. Young’s elastic modulus (*E*) is a measure of a material’s stiffness, defining the relationship between applied force and deformation, and is generally measured in units of Pascals (Pa). Elasticity can be calculated by fitting data collected from indentation measurements and is an intrinsic biomechanical property. Nanoindentation data were recorded as force vs. distance curves (Figure 5E and Supplementary Figure 2), and Young’s elasticity moduli (*E*) were calculated as outlined in Collett et al. (2019). Mean *E* values ranged from 6.2 (DEN-2 VLP) to 21.3 MPa (DEN-4 VLP) (Figure 5F and Table 2). Young’s modulus data were analysed as for height and diameter AFM data, with significant differences found between DEN VLPs from all serotypes by both ANOVA and Kruskal-Wallis tests. Pairwise comparisons performed using the Mann–Whitney test show a statistically significant difference (*p* < 0.01) in elasticity between all serotypes (Figure 5F). Elasticity values obtained for DEN VLPs fall within the range of values obtained from other VLPs derived from enveloped viruses (Collett et al., 2019). Unfortunately, insufficient data could be collected from SARS-CoV-2 particles to determine elasticity.

**Table 2:** Mean Young’s elasticity (E), with standard deviation, for all DEN VLP serotypes.

Serotype	Mean E value (MPa)	Std. dev.
DEN-2 (*n* = 180)	6.2	5.2
DEN-3 (*n* = 99)	9.0	7.4
DEN-4 (*n* = 112)	21.3	13.7

VLPs produced from different DEN serotypes were found to have different sizes and elasticities. However, particle stiffness (elasticity) did not necessarily correlate with particle size (Figure 5 and Tables 1, 2).

### 3.6. α-GalCer-adjuvanted VLPs enhance NKT cell immune responses *in vitro*

To improve immune responses to the VLPs, we developed a novel method to produce self-adjuvanted DEN and SARS-CoV-2 VLPs, by the incorporation of the CD1d-restricted NKT-cell agonist α-GalCer within the VLP manufacturing process (Figure 1C). This method was shown not to interfere with the production of E of DEN-2 or the S protein of SARS-CoV-2, as shown by Western blot of purified VLPs produced with increasing amounts of α-GalCer (Figures 2H, 3C).

**Figure 6:**
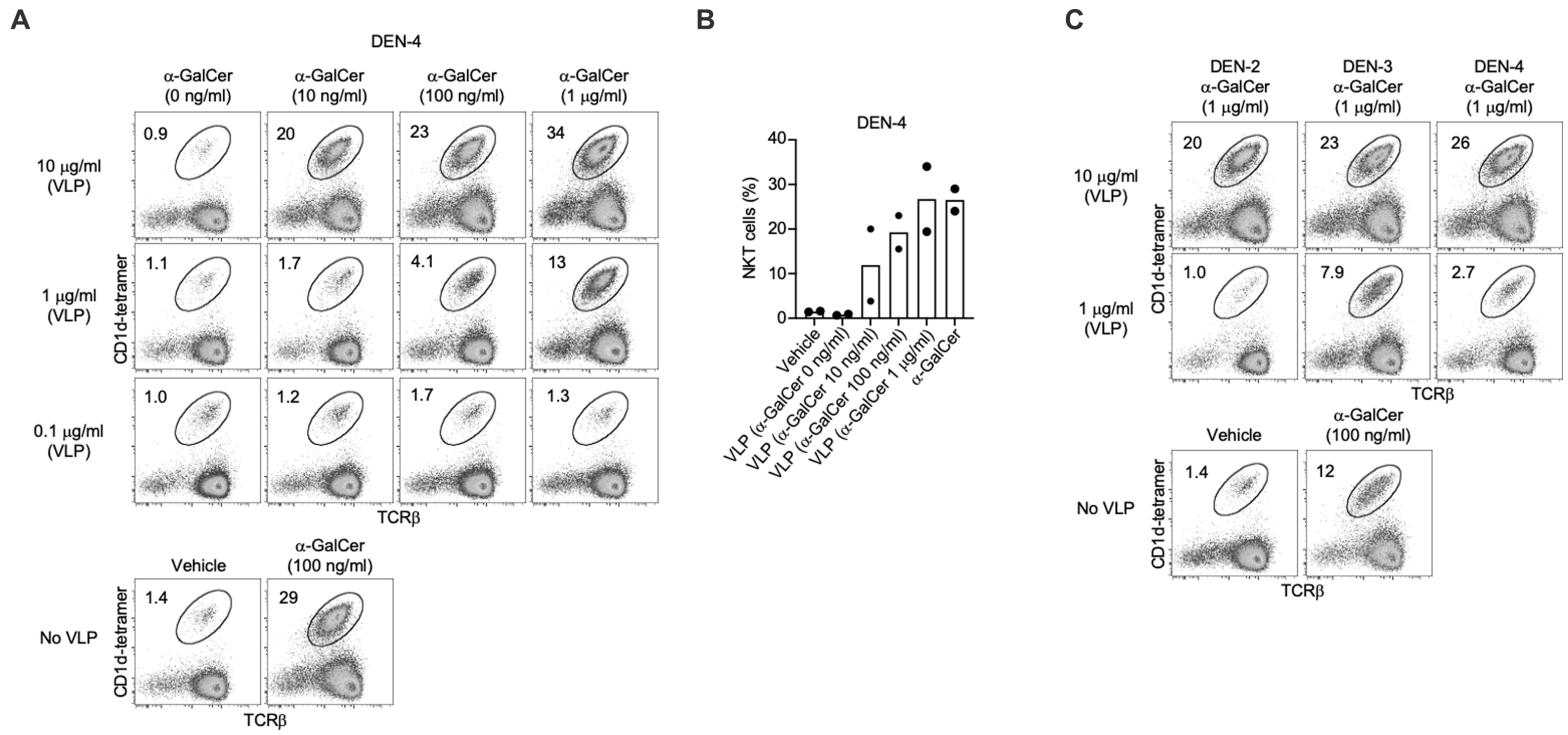
NKT cell activation induced by self-adjuvanted DEN VLPs. Splenocytes (C57BL/6) were stimulated for 4 days *in vitro* under the indicated condition prior to FACS analysis. Numbers on plots indicate the percentage of NKT cells amongst viable (7-AAD-), CD19- lymphocytes. **(A)** Dose response of DEN-4 VLPs generated with differing concentrations of α-GalCer. **(B)** Data pooled from two independent experiments at 10 µg/mL VLP dose. **(C)** Comparison of NKT cell expansion using VLPs from the indicated serotype, shown in comparison to α-GalCer alone and vehicle controls from 1 experiments (see Supplementary Figure 3).

Inclusion of α-GalCer within DEN-4 VLPs was confirmed by assessing NKT cell expansion following *in vitro* splenocyte cultures (Figures 6A,B), with the proportion of NKT cells correlating with the concentration of α-GalCer added during the VLP production process. Although 10 μg/mL of VLPs produced with 100 ng/mL of α-GalCer led to a slightly lower level of NKT cell expansion than the control of 100 ng/mL of α-GalCer delivered straight to the cells (Figures 6A,B), this is not surprising as less of the α-GalCer would be available to stimulate cells when incorporated into VLPs. The incorporation of α-GalCer into DEN-2, −3, and −4 VLP serotypes also resulted in a strong expansion of NKT cells (Figure 6C). At an *in vitro* concentration of 10 μg/mL, the extent of NKT cell expansion was similar from each DEN VLP serotype. However, the α-GalCer-DEN-3 VLP formulation provided the most potent NKT cell increase at the lower dose of 1 μg/mL (Figure 6C) and so DEN-3 VLPs were chosen for subsequent *in vivo* studies. NKT cell expansion by the α-GalCer-DEN-3 VLP formulation was confirmed in a separate experiment (Supplementary Figure 3).

**Figure 7:**
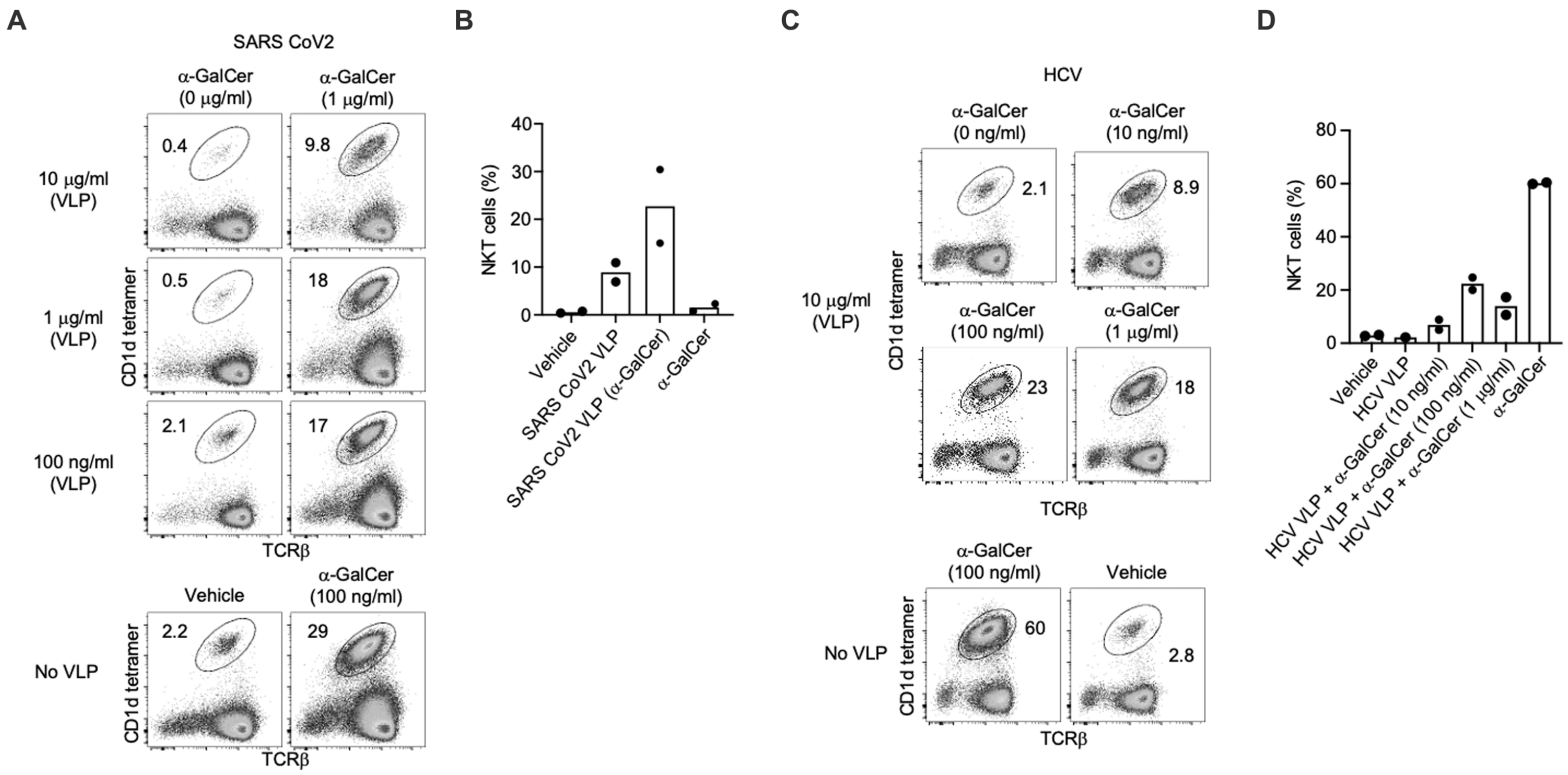
NKT cell activation induced by self-adjuvanted HCV and SARS CoV-2 VLPs. Splenocytes (C57BL/6) were stimulated for 4 days *in vitro*. Numbers on FACS plots indicate the percentage of NKT cells amongst viable (7-AAD-), CD19- lymphocytes from **(A)** SARS CoV-2 or **(C)** HCV VLPs generated in the presence of the indicated dose of α-GalCer shown in comparison to α-GalCer alone or vehicle controls. Pooled data of NKT cells percentages following *in vitro* culture with **(B)** SARS CoV-2 or **(D)** HCV VLPs. Data sourced from three independent experiments.

Similarly, α-GalCer-SARS-CoV-2 VLPs activated strong expansion of NKT cells *in vitro* (Figures 7A,B). As further evidence the observed NKT expansion was not specific to VLPs derived from a particular virus, we manufactured HCV-1a VLPs, previously developed in our labs (Earnest-Silveira et al., 2016a,b; Christiansen et al., 2018a,b) incorporating α-GalCer within the VLP manufacturing process. For HCV-1a VLPs, the strongest expansion of NKT cells was detected using VLPs produced with 100 ng/mL of α-GalCer (Figures 7B,C).

### 3.7. *In vivo* immunogenicity of self-adjuvanted VLPs

**Figure 8:**
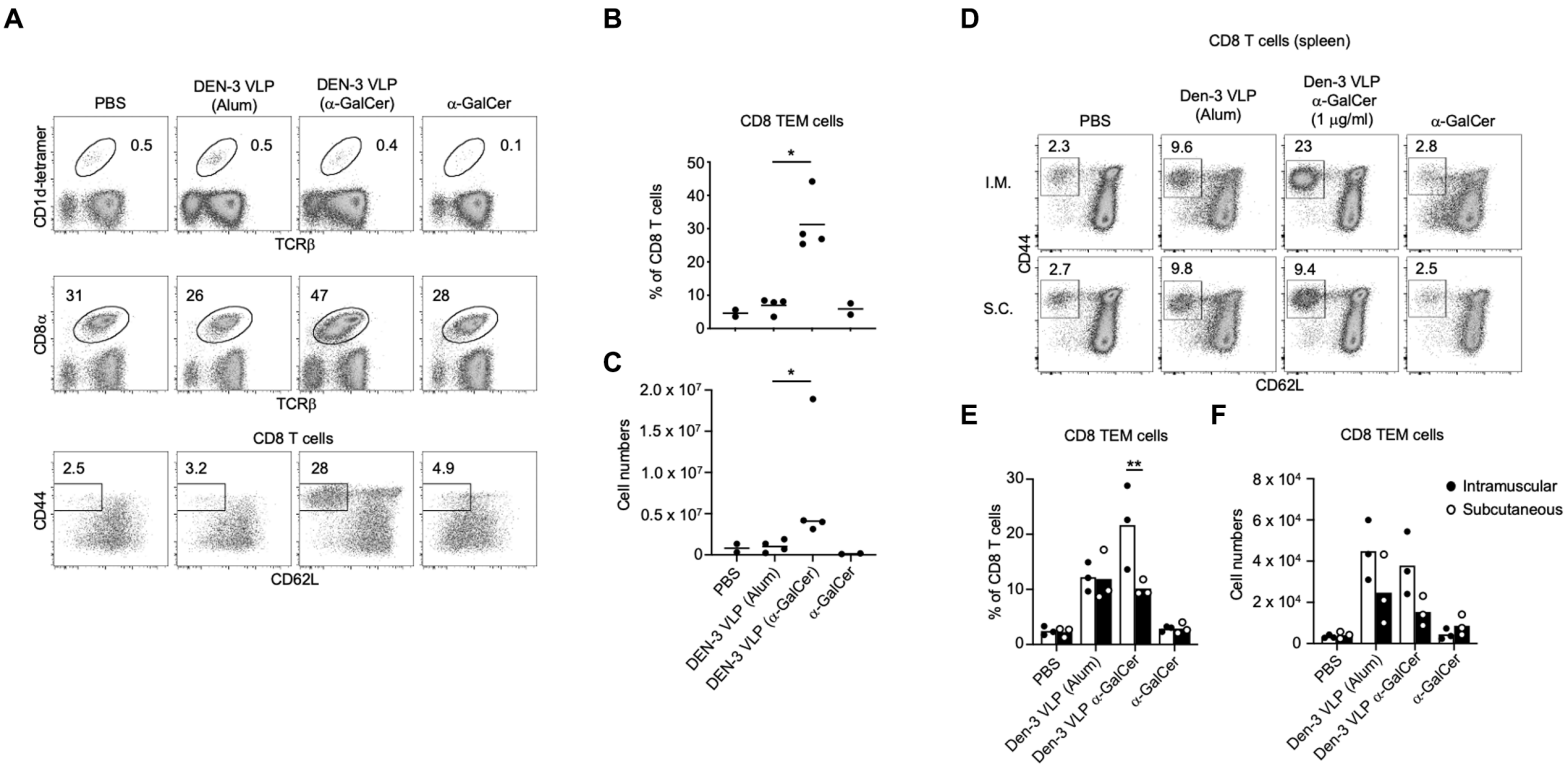
Self-adjuvanted DEN VLPs elicit CD8 effector memory T cell responses. C57BL/6 mice were immunised subcutaneously with two doses of α-GalCer-DEN-3 VLP, DEN-3 VLP (delivered with Alum), α-GalCer alone or PBS. At day 21, spleens were harvested and analysed by FACS. **(A)** Representative plots showing the percentage of NKT cells (top) or CD8 T cells (middle) amongst viable (7-AAD-), CD19- lymphocytes. Bottom row depicts the percentage of CD8+ effector-memory (Tem) cells (CD44+ CD62L-) within the CD8 T cell population. **(B)** Graph depicts the percentage of CD8 Tem cells amongst CD8 T cells. **(C)** Represent the number of splenic CD8 Tem cells. Each point represents one animal with the bar showing the mean **(B,C)**. Statistical significance between VLP (Alum) and VLP (α-GalCer) assessed via a two-tailed Mann Whitney test (*p < 0.05). **(D)** C57BL/6 mice were immunised either intramuscularly or subcutaneously with two doses of α-GalCer-DEN-3 VLP, DEN-3 VLP (delivered with Alum), α-GalCer alone or PBS. At day 42, spleens were harvested and analysed by FACS. **(E)** Graph depicts the percentage of CD8 TEM cells amongst CD8 T cells. **(F)** Statistical significance of injection route was assessed via way a two-way ANOVA with Bonferroni post-test correction (**p < 0.01).

We next determined whether the incorporation of α-GalCer into VLPs resulted in vaccines with enhanced immunogenicity *in vivo*. As proof of principle, we examined *in vivo* T cell and NKT cell responses to self-adjuvanted DEN-3 VLPs, and B cell and antibody responses to self-adjuvanted DEN-3, SARS-CoV-2 and HCV-1a VLPs. To determine whether the self-adjuvanted DEN VLPs were immunogenic *in vivo*, we immunised mice with two doses of 10 μg of α-GalCer-DEN-3 VLP, subcutaneously or intramuscularly, 2 weeks apart. One week after the boost, spleens were harvested for FACS analysis. Despite our short-term *in vitro* experiments demonstrating increased splenic NKT cell percentages in response to α-GalCer-DEN-3 VLPs (Figure 6C), NKT cell expansion was not evident from *in vivo* inoculated mice (Figure 8A and Supplementary Figure 3), which is consistent with the rapid contraction of the NKT cell population reported following the *in vivo* administration of α-GalCer (Crowe et al., 2003; Uldrich et al., 2005). However, the proportion of CD8 T cells amongst CD19- lymphocytes was markedly enhanced in mice immunised with α-GalCer-DEN-3 VLPs compared to those injected with DEN-3 VLP (delivered with Alum), α-GalCer alone or the vehicle control (Figure 8A). Notably, this appeared to result from the expansion of CD8^+^ CD44^+^, CD62L^−^ effector memory T (Tem) cells, from less than 10% with VLP adjuvanted with Alum to approximately 30% of CD8 T cells when α-GalCer was incorporated into the VLP (Figures 8B,C). Furthermore, inoculation of mice with intramuscular α-GalCer-DEN-3 VLPs resulted in a greater CD8^+^ Tem cell response when using the intramuscular route than with the subcutaneous route (Figures 8D–F).

**Figure 9:**
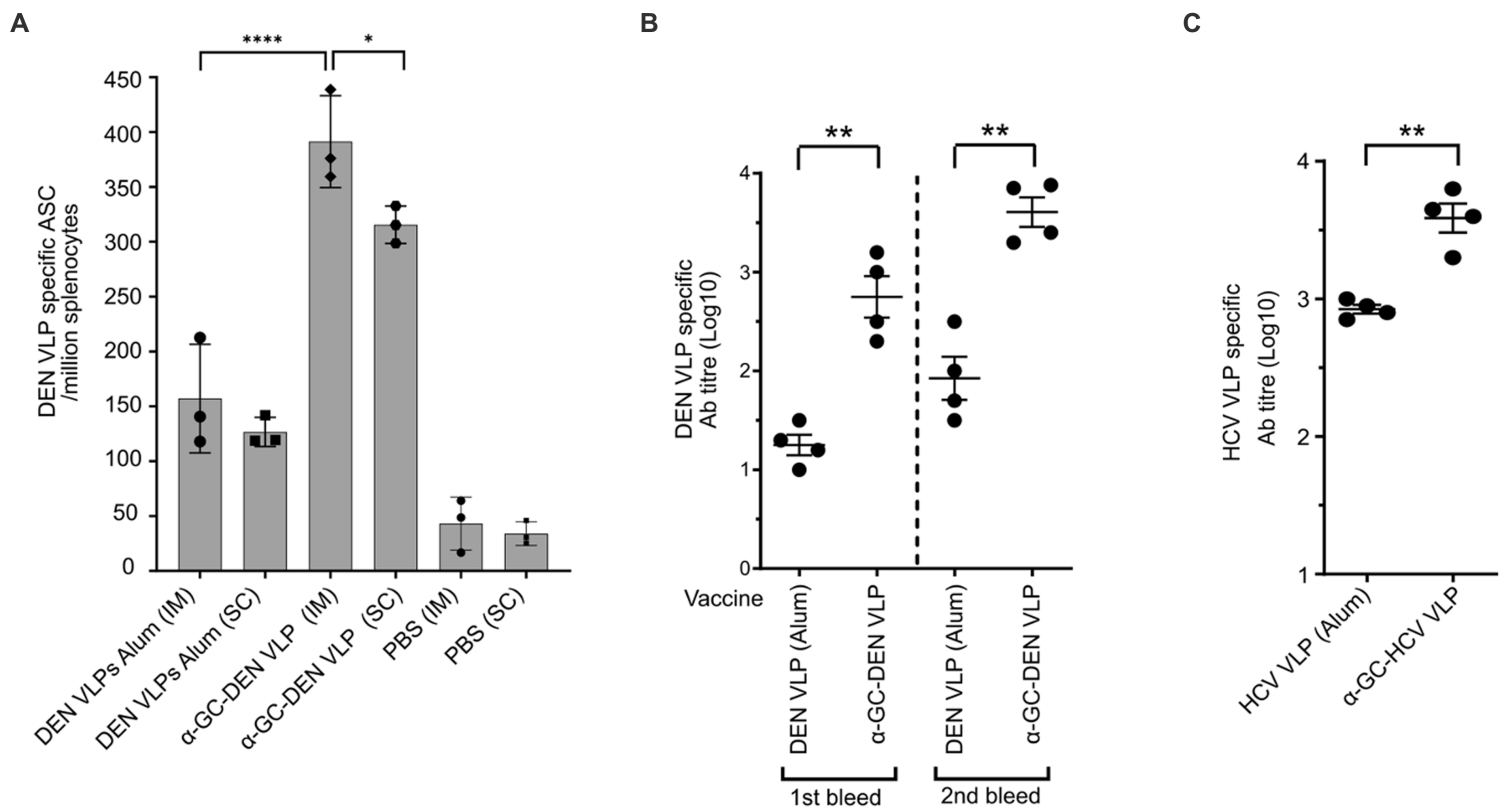
B cell and antibody responses following VLP vaccination with α-GalCer self-adjuvanted DEN and HCV VLPs compared with VLPs adjuvanted with Alum. **(A)** B cell numbers after intra-muscular (IM) vs. subcutaneous (SC) delivery, and **(B)** end-point antibody titres after a single booster dose of α-GalCer-DEN-3 VLP. **(C)** End-point antibody titres after vaccination with self-adjuvanted, as compared to non-adjuvanted, HCV VLPs. B cell numbers were determined by ELISpot assay and antibody titers were determined by ELISA. ELISA plates were coated with VLP alone at 20 μg/mL. Endpoint titres were determined by establishing cutoff values from pre-inoculation samples. One-way ANOVA tests were used to determine statistical significance (*p* < 0.05, * < 0.05, ** < 0.005, *** < 0.0005, **** < 0.0001).

To investigate B cell responses to these self-adjuvanted VLP vaccines, vaccinated mice were bled after the first and second dose and B-cells and antibody titers measured by B-cell ELISPOT and ELISA, respectively. Although strong antibody responses were seen after only one dose of vaccine, antibody titers increased after a single booster administered 14 days after the initial immunisation. Importantly, mice vaccinated with α-GalCer-DEN-3 VLP developed significantly stronger antibody and B-cell responses than mice vaccinated with DEN-3 VLP adjuvanted with Alum (Figures 9A,B). A similar increase in antibody response was seen in mice immunised with α-GalCer-HCV-1a VLP compared to HCV-1a VLP adjuvanted with Alum (Figure 9C). Further, as with the Tem cell responses, B cell responses were found to be significantly higher in animals inoculated through the intramuscular route (Figure 9A).

Immune responses in immunised mice were investigated for SARS-CoV-2 VLPs, delivered without adjuvant, with the commercial adjuvant AddaVax, and as our self-adjuvanted α-GalCer-VLP formulation. AddaVax is a research grade squalene-based adjuvant equivalent to the licensed MF59 and was used for the SARS-CoV-2 VLPs as this work is currently directed toward producing a licensed COVID-19 vaccine. On the back of successful influenza vaccines delivered with MF59, we were interested to see if vaccines against other respiratory viruses would benefit from delivery with AddaVax, and how that compares to our self-adjuvanted VLP formulation.

**Figure 10:**
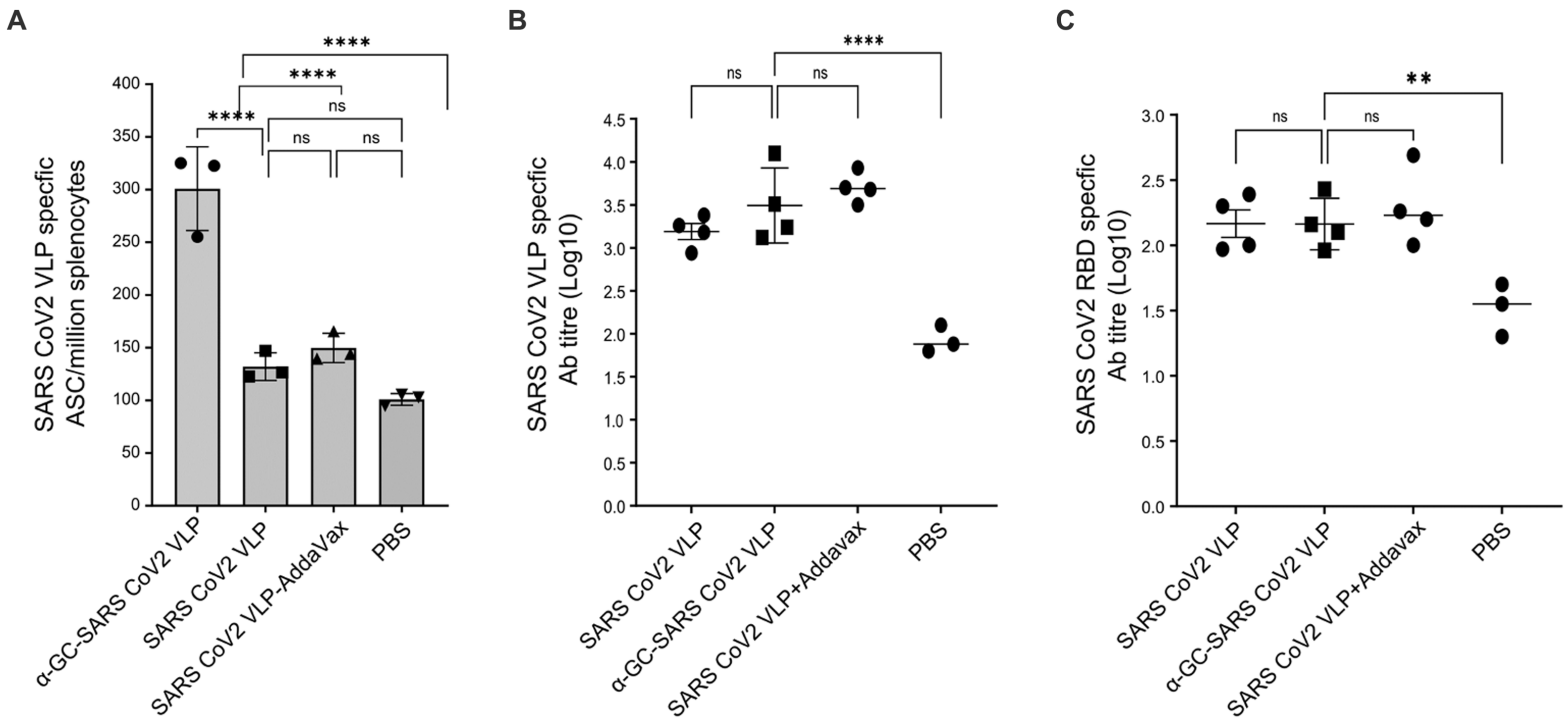
Immediate B cell and antibody responses following SARS-CoV-2 VLP vaccination. C57BL/6 mice were immunised intramuscularly with 20 μg of either SARS-CoV-2 VLP, α-GalCer-SARS-CoV-2 VLP or SARS-CoV-2 VLP delivered with AddaVax, or PBS alone as control, at days 0 and 7. Mice were harvested on day 10. **(A)** Total SARS-CoV-2 specific B cells per million splenocytes, as determined by ELISpot assay. **(B)** SARS-CoV-2 VLP specific antibody titre, and **(C)** SARS-CoV-2 RBD specific antibody responses in immunised mouse sera, as measured by ELISA assay. ELISA plates were coated with VLP at 20 μg/mL or RBD at 5 μg/mL. Each point represents one animal with bars showing the mean. Endpoint titres were determined by establishing cutoff values from pre-inoculation samples. One-way ANOVA tests were used to determine statistical significance (*p* < 0.05, * < 0.05, ** < 0.005, *** < 0.0005, **** < 0.0001).

In mice immunised at days 0 and 7, and examined at day 10, immediate SARS-CoV-2 VLP specific B cell responses were found to be significantly higher in the group immunised with the α-GalCer-VLP formulation than in those immunised with either VLP alone or VLP plus AddaVax. No significant difference in B cell activation was found between these two groups and the PBS control group at day 10 (Figure 10A). However, antibody titres were found to be significantly higher in all three immunisation groups at this timepoint compared to the PBS control for both SARS-CoV-2 VLP specific antibodies (Figure 10B) and for SARS-CoV-2 RBD specific antibodies (Figure 10C). These results also showed that the antibody responses produced with α-GalCer-VLPs were not inferior to VLP plus AddaVax.

**Figure 11:**
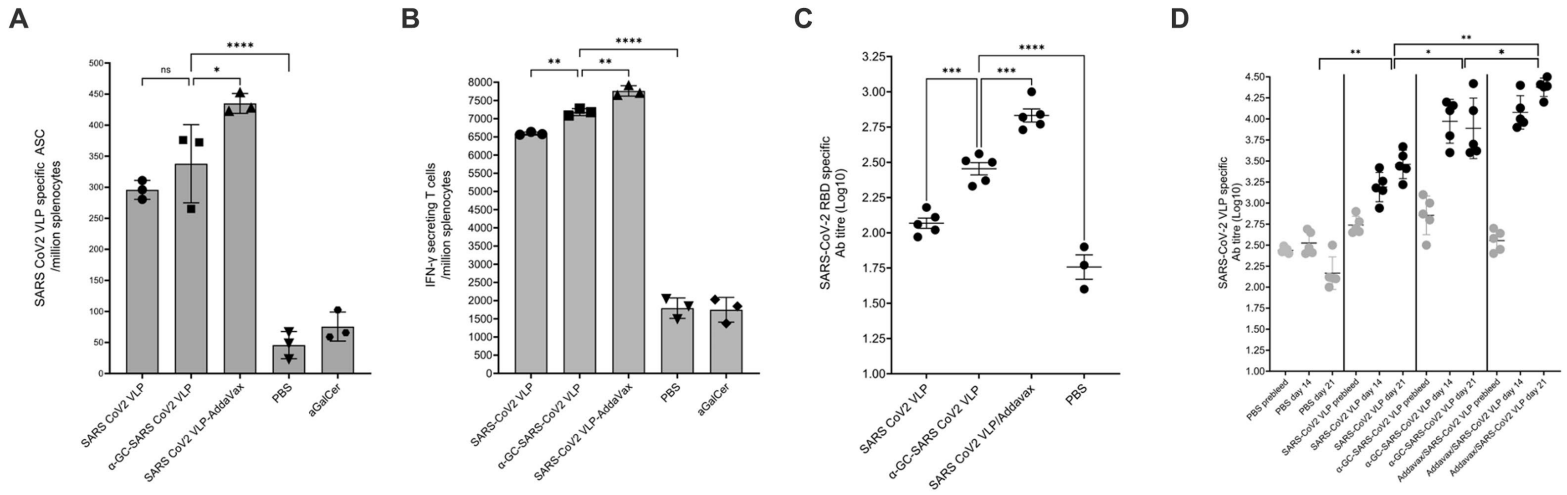
B cell and antibody responses following SARS-CoV-2 VLP vaccination. C57BL/6 mice were immunised intramuscularly with 20 μg of either SARS-CoV-2 VLP, α-GalCer-SARS-CoV-2 VLP or SARS-CoV-2 VLP delivered with AddaVax, or PBS alone as control, at days 0 and day 14, bled at days 0 and 14 and harvested on day 21. **(A)** Total SARS-CoV-2 specific B cells per million splenocytes, as determined by ELISpot assay. **(B)** Total IFN-γ secreting cells per million splenocytes, as determined by ELISpot assay. **(C)** SARS-CoV-2 VLP specific antibody titre at day 21, as determined by ELISA assay. **(D)** SARS-CoV-2 VLP specific antibody titre at days 0, 14 and 21, as determined by ELISA assay. ELISA plates were coated with VLP at 20 μg/mL. Each point represents one animal with bars showing the mean. Endpoint titres were determined by establishing cutoff values from pre-inoculation samples. One-way ANOVA tests were used to determine statistical significance (*p* < 0.05, * < 0.05, ** < 0.005, *** < 0.0005, **** < 0.0001).

In mice immunised on days 0 and 14 and examined on day 21, VLP specific B cell responses were found to be significantly higher in the group receiving the VLP plus AddaVax, with no significant difference found between the α-GalCer-VLP formulation and VLP alone. All vaccinated mice showed significantly higher B cell responses than controls (Figure 11A). At this timepoint, IFN-γ secreting T cells were highest in mice inoculated with VLP plus AddaVax, but significantly higher in the α-GalCer-VLP formulation group than in mice that received VLP alone (Figure 11B), with the same trend seen in SARS-CoV-2 VLP specific antibody titres (Figures 11C,D).

**Figure 12:**
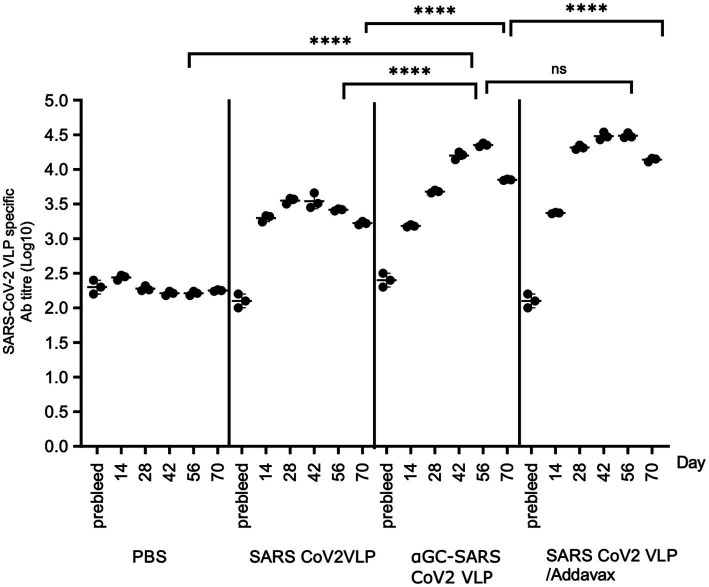
Sustained antibody responses following SARS-CoV-2 VLP vaccination. SARS-CoV-2 VLP specific antibody titre, as determined by ELISA assay. C57BL/6 mice were immunised intramuscularly with 20 μg of either SARS-CoV-2 VLP, α-GalCer-SARS-CoV-2 VLP or SARS-CoV-2 VLP delivered with AddaVax, or PBS alone as control, at day 0 and day 14, bled at day 0, 14, 28 and 56, and harvested on day 70. ELISA plates were coated with VLP at 20 μg/mL. Each point represents one animal with bars showing the mean. Endpoint titres were determined by establishing cutoff values from pre-inoculation samples. One-way ANOVA tests were used to determine statistical significance (*p* < 0.05, **** < 0.0001).

We next examined the durability of antibody responses out to 70 days in mice immunised on days 0 and 14, with antibody titres determined on days 14, 28, 42, 56, and 70. Significantly higher SARS-CoV-2 VLP specific Ab titres were seen in all the VLP delivery regimens compared to PBS controls. At 28 days, Ab titres in the AddaVax group were significantly higher than the α-GalCer-VLP group. However, at 56 days (peak Ab titres), no significant difference in Ab titre was seen in mice immunised with α-GalCer-VLPs compared with AddaVax adjuvanted VLPs, and Ab titres were significantly higher in both α-GalCer-VLP formulation and VLP delivered with AddaVax compared to non-adjuvanted VLP. All groups saw a drop in Ab titres at the 70-day point, with titres in the α-GalCer-VLP falling to levels significantly below the AddaVax group, although titres remained above day 14 levels and well above pre-bleed levels (Figure 12). This may indicate the need for further boosting with the α-GalCer-VLP formulation. While the α-GalCer-VLP formulation was not found to generate better B cell and IFN-γ secreting T cell responses than VLP delivered with AddaVax, peak Ab titres were statistically equivalent. Whether the self-adjuvanted SARS-CoV-2 VLP formulation can elicit higher TEM responses than traditional adjuvants, as was seen with the DEN and HCV VLPs, is currently being investigated in our laboratory.

## 4. Discussion

The most effective vaccines in clinical use include attenuated viral, inactivated, subunit and synthetic conjugate vaccines (Carreno et al., 2014; Liu and Guo, 2017), as well as the more recent developments in mRNA vaccines (Chakraborty et al., 2021). However, the ability of these vaccines to stimulate potent long-term protective and memory immune responses may be limited. To overcome this problem, vaccines are often delivered with adjuvants that act as immunostimulants. We have developed VLPs of SARS-CoV-2, DENV 2, 3 and 4 and previously HCV (genotypes 1 to 4) (Earnest-Silveira et al., 2016a,b; Kumar et al., 2016; Christiansen et al., 2018a,b, 2019) that contain the major viral structural proteins. The novelty of our approach entails the inclusion of a SP/SPP sequence separating individual structural proteins to ensure that a viral polyprotein will cleave intracellularly, thereby releasing individual structural proteins that are then able to assemble into VLPs. Consequently, by allowing multiple proteins to be delivered in a single construct, our approach facilitates the production of VLPs at large scale. We have also shown our VLPs can be produced as self-adjuvanted vaccine candidates with enhanced immunogenicity in a small animal model.

Eukaryotic membrane and secretory proteins contain short signal sequences that are required for targeting proteins to the endoplasmic reticulum (ER) and ultimately for entry into the secretory pathway (Lemberg and Martoglio, 2002). After insertion of a protein into the ER membrane, signal peptides are cleaved from the precursor protein by a signal peptidase. Signal peptides that span the ER membrane can then be further cleaved by the presenilin-type aspartic protease signal peptide peptidase (SPP) thereby releasing the protein from the ER membrane. Cleavage of the signal peptide by signal peptidase at the exoplasmic side of the ER is essential before SPP is able to cleave within the transmembrane region to release the cleavage product (Lemberg and Martoglio, 2002). Intramembrane proteolysis by SPP promotes the release of signal peptide fragments and proteins from the ER membrane (Weihofen et al., 2002).

Hepatitis C utilises SPP for the processing of the viral polyprotein (10–13). A short hydrophobic sequence separating the viral core and envelope proteins targets the nascent polyprotein to the ER where the polyprotein undergoes cleavage by signal peptidase followed by intramembrane SPP cleavage to release the core protein which is then able to migrate to the replication complex for viral assembly. This two-step cleavage is unique to HCV in contrast to other positive sense RNA viruses such as alpha-, flavi- and rubiviruses (McLauchlan et al., 2002). Exploiting cellular SP/SPP rather than a viral protease-dependent cleavage has enabled us to create polyproteins of several RNA viruses containing the major structural proteins that undergo cell-dependent cleavage in the ER thereby allowing these proteins to self-assemble into VLPs.

VLP vaccines have the potential to induce strong protective immune responses and the lack of genetic material in VLPs also negates the risk of reversion to virulence, thereby endowing VLP vaccines with a better safety profile than live attenuated vaccines while retaining a high level of immunogenicity (Oussoren et al., 1998; Reddy et al., 2007; Manolova et al., 2008; Braun et al., 2012; Zabel et al., 2014). Further, VLPs are better suited than most nanoparticle formulations to present multiple, and conformationally dependent, epitopes (Oscherwitz, 2016), thereby potentially leading to higher affinity antibody production. Additionally, sequences can easily be matched to circulating strains to readily produce VLP vaccines to viral variants. Introducing the SP/SPP cell-dependent cleavage sequence between individual structural viral proteins in a polyprotein construct provides an approach that can facilitate the production of VLPs as viral vaccines.

The ability to also produce self-adjuvanting VLPs that are made of the major viral structural proteins and incorporate a glycolipid adjuvant represents a further advance in vaccine technology. Exposure to α-GalCer activates NKT cells that stimulate the development of cytotoxic and memory T cell, and antibody responses. Activated NKT cells can help to activate multiple immune cell types including conventional T cells, B cells, and dendritic cells (Oscherwitz, 2016). The application of α-GalCer as a potential vaccine adjuvant has therefore generated considerable interest (48–52). Strategies to develop NKT activating vaccines could therefore have several advantages by producing broad protective immune responses compared with standard vaccines (Carreno et al., 2014; Liu and Guo, 2017). Several approaches to conjugate or incorporate glycolipids to vaccine antigens (Liu and Guo, 2017) have also been developed to optimise the delivery of glycolipid adjuvants and reduce the potential for NKT anergy and toxicity (Carreno et al., 2014). Our vaccine platform is a significant advance in vaccine research as we have developed a novel method to incorporate glycolipid adjuvants directly into VLPs during intracellular particle self-assembly.

We have previously shown that HCV VLPs representing the major genotypes and incorporating SP/SPP cleavage can be produced to large scale and are immunogenic in a large animal model (Earnest-Silveira et al., 2016a,b; Kumar et al., 2016; Christiansen et al., 2018a,b, 2019). In this study we have shown that α-GalCer-HCV VLPs of genotype 1a produced up to a 10-fold increase in NKT activation that was accompanied by a stronger antibody response *in vivo* than VLPs adjuvanted with alum.

More extensive studies were performed with the DEN VLPs to provide further proof of concept for producing self-adjuvanted SP/SPP cleaving VLPs. Our α-GalCer-DEN VLPs produced strong activation of NKT cells *in-vitro*. In contrast to DEN VLPs adjuvanted with alum, vaccination of mice with α-GalCer-DEN-3 VLPs produced strong antibody, B cell and memory CD8^+^ T cell responses, particularly following intramuscular inoculation compared to subcutaneous administration. We provide proof of principle that this approach induces strong antibody and T cell responses and further investigation is warranted. However, these results highlight the potential of α-GalCer-DEN VLPs as a vaccine candidate and may help to overcome some of the challenges faced by current chimeric and live attenuated DEN vaccines in producing a balanced immune response against 4 serotypes and in some cases requiring different dosing for each serotype in order to achieve this (Capeding et al., 2014; Hadinegoro et al., 2015; Villar et al., 2015; Biswal et al., 2019, 2020; López-Medina et al., 2020; Rivera et al., 2021).

For DENV, the most potent NAbs in humans are directed to EDE1 and EDE2 of the envelope protein dimer (Dejnirattisai et al., 2015; Gallichotte et al., 2015), and include conformational epitopes only present on intact virions (de Alwis et al., 2012; Fibriansah et al., 2014, 2015). Identification of critical neutralisation domains is further complicated by masking of epitopes due to viral envelope plasticity (Lok et al., 2008; Fibriansah et al., 2013). We were limited in gaining access to a broader range of HuMAbs, however, our DEN VLPs bound HuMAbs known to bind the conformationally dependent, neutralising epitopes on EDE1 and EDE2. The inclusion of the DENV capsid (C) protein in our VLP formulation may be important in achieving this (Lazo et al., 2010). These observations strengthen the argument for developing VLP vaccines capable of presenting conformationally authentic antigen.

Boigard et al. (2018) reported the generation of DEN-2 VLPs containing the DENV-2 structural proteins C/prM/E, as well as NS2B and the viral protease NS3 to allow for capsid protein cleavage. They used the same three point mutations in the E protein we utilised in our study but produced their VLPs using co-transfection with separate DNA plasmids (Purdy and Chang, 2005; Hsieh et al., 2008). An important advantage of our approach is the inclusion of the SP/SPP sequence to allow cleavage and release of the capsid protein thereby avoiding the need to include the DENV NS3/2b complex which can potentially interfere with endogenous cellular IFN signalling pathways (Anglero-Rodriguez et al., 2014).

Finally, it may be desirable for a DENV vaccine to elicit strong CD4^+^, CD8^+^ T cell and innate immune responses (Dejnirattisai et al., 2010; Rivino et al., 2013; Barba-Spaeth et al., 2016; Elong Ngono et al., 2016; Rivino and Lim, 2017; Robbiani et al., 2017). To enhance the immunogenicity of the DENV VLP vaccines, we developed an approach whereby the glycolipid adjuvant α-GalCer is incorporated into the VLP. This novel self-adjuvanting strategy has allowed us to produce VLPs from HCV, DENV and SARS-CoV-2 that can stimulate NKT cells, harnessing their adjuvant like effects *in vivo* and, for the DEN VLPs, induce a strong Tem response.

With the onset of the COVID-19 pandemic, and as a further example of our SP/SPP self-adjuvanted vaccine platform, we developed SARS-CoV-2 VLPs. These particles assembled into VLPs that are morphologically analogous to SARS-CoV-2 virus with characteristic spikes. Importantly, SARS-CoV-2 VLPs produced in the presence of α-GalCer stimulated strong NKT responses *in vitro*. Further, our self-adjuvanted α-GalCer-SARS-CoV-2 VLP vaccine induced sustained antibody responses in vaccinated mice, with peak Ab titres equivalent to those induced by VLPs adjuvanted with the licensed adjuvant AddaVax.

A further advantage of our approach is that the S protein can be interchanged with the S of emerging variants to produce a vaccine that is more broadly protective against current circulating viruses. Since the start of the COVID-19 pandemic, SARS-CoV-2 has evolved relatively rapidly to into a large family of variants that has been referred to as a ‘variant swarm’ or ‘variant soup’ (Callaway, 2022). The current vaccines have relied on the delivery of the S protein or mRNA, or the RBD, to activate S-specific humoral immune responses (Ewer et al., 2021; Heath et al., 2021). However, emerging variants have proven to be more infectious and capable of evading NAb that are directed to the RBD and S protein (Weisblum et al., 2020). Omicron variants are rapidly evolving with increasing ability to escape vaccine-induced NAb, with studies showing that neutralisation of early Omicron variants by vaccinee immune sera is reduced 20- to 40-fold (Dejnirattisai et al., 2022; Pajon et al., 2022). Boosting with BNT162b2 or mRNA-1273 vaccines increases NAb titres to these variants, albeit at a lower level than to ancestral Wuh1 virus and other variants including Delta (Doria-Rose et al., 2021; Dejnirattisai et al., 2022; Nemet et al., 2022; Pajon et al., 2022). However, the more recent Omicron descendants, BQ1.1 and XBB.1, almost completely escape vaccine induced immunity and have been responsible for resurgent epidemic waves (Callaway, 2022; Kurhade et al., 2022; Uraki et al., 2022).

Vaccination of previously infected individuals provides a high level of protection against reinfection with many SARS-CoV-2 variants (Bozio et al., 2021). However, for naïve individuals, three doses of BNT162b2 mRNA vaccine is required to protect against Beta and Delta SARS-CoV-2 (Falsey et al., 2021) producing an 86% reduction in the risk of reinfection and up to a 97% reduction in hospitalisation (Patalon et al., 2021). For the more recent Omicron variants, a 4th dose of a mRNA vaccine is helpful (Regev-Yochay et al., 2022) or alternatively, vaccination with a BA.5 containing bivalent mRNA vaccine can boost immune response against Omicron BQ1.1 and to some extent against XBB.1 (Kurhade et al., 2022). Memory CD4+ and CD8+ T cells induced after vaccination with ancestral SARS-CoV-2 vaccines (mRNA-1273, BNT162b2, Ad26.COV2.S, and NVX-CoV2373) are cross-reactive to Alpha, Beta, Delta, Gamma and Omicron and these responses are preserved for at least 6 months post vaccination (Tarke et al., 2022). These findings may help to explain why current SARS-CoV-2 vaccines protect against severe disease following infection with SARS-CoV-2 variants such as Omicron but also highlight the importance of developing vaccines that are able to activate CD4+ and CD8+ T cell responses in addition to NAb (Ferguson et al., 2021). SARS-CoV-2 VLPs that include the major structural proteins have the potential to fulfil this requirement.

What is becoming apparent is that mRNA vaccines provide relatively short-lived immunity and as variants become more antigenically distant to the Wuh1 virus and earlier variants, vaccines based on the ancestral S sequence have become much less effective. An approach like ours using self-adjuvanted VLPs that can be produced at large scale may provide an alternative to current vaccines. We are now developing our SARS-CoV-2 VLPs further to include Beta and Omicron lineage S proteins as next generation booster vaccines with the potential to produce broad protective immune responses that may also be more durable.

## 5. Conclusion

We have produced VLPs of SARS-CoV-2 and DENV using a unique approach incorporating SP/SPP cellular cleavage of a viral polyprotein containing the major structural proteins and thereby allow for efficient self-assembly of proteins into VLPs. We characterised the VLPs biochemically, confirming the presence of C and E proteins in DEN VLPs and of S, E and M in SARS-CoV-2 VLPs. The morphology of VLPs was characterised using TEM and AFM. An important part of our process was the development of methods for large scale laboratory production and purification of the VLPs that are also adaptable for industry manufacturing. A further advantage of our approach is the development of self-adjuvanted VLP vaccines. We showed that our α-GalCer-VLP formulation with DEN, HCV and SARS-CoV-2 VLPs activated NKT cells *in vitro*. Further, our α-GalCer-DEN VLP vaccine produced strong effector memory CD8^+^ T cell responses after only two inoculations of vaccine. Administration of the self-adjuvanted DEN and HCV VLPs *in vivo* was associated with a significantly stronger antibody and CD8^+^ TEM response compared to VLPs delivered with Alum. Inoculation of mice with self-adjuvanted SARS-CoV-2 VLPs induced strong T cell, B cell and antibody responses, although these were generally seen to be lower than responses induced by SARS-CoV-2 VLPs delivered with AddaVax. Peak Ab titres, however, were found to be equivalent between SARS-CoV-2 VLPs delivered with AddaVax and our self-adjuvanted VLPs. Protection was not evaluated in this study. However, work developing and assessing the SARS-CoV-2 VLPs is ongoing in our laboratory. Our novel self-adjuvanted SP/SPP cleaving VLP vaccines have the potential to produce broad cross-protective responses and are amenable for development as vaccine candidates for other enveloped RNA viruses.

## Data availability statement

The original contributions presented in the study are included in the article/Supplementary material, further inquiries can be directed to the corresponding author.

## Ethics statement

The animal study was reviewed and approved by the University of Melbourne Animal Ethics Committee.

## Author contributions

SC: writing—original draft, investigation, validation, methodology, formal analysis, data curation, and visualisation. LE: investigation, validation, methodology, formal analysis, and data curation. JC, AY, and JL: investigation. ME and SM: investigation and writing—review and editing. CW, VL, and VP: writing—review and editing. DC: investigation, validation, methodology, and formal analysis. JR and JM: investigation, validation, and methodology. CS: conceptualization, validation, and writing—review and editing. SG and DH: conceptualization and writing—review and editing. JMM: conceptualization, validation, and resources review editing. AE and PR: resources, writing—review and editing, and supervision. GC: investigation, validation, formal analysis, data curation, writing—review and editing, and visualisation. DG: conceptualization, validation, resources, and writing—review and editing. JT: writing—original draft, conceptualization, validation, resources, supervision, project administration, and funding acquisition. All authors contributed to the article and approved the submitted version.

## Funding

This work was supported by a grant from NHMRC MRFF 2020 COVID-19 Vaccine Candidate Research, Australia (APP2013957) and an unrestricted research grant from Sanofi Pasteur. The research was performed in part at the RMIT Micro Nano Research Facility (MNRF) in the Victorian Node of the Australian National Fabrication Facility (ANFF). The Cypher ES AFM instrument was funded in part by grant LE170100096 from the Australian Research Council (ARC). SC was supported by a research training program stipend scholarship from the Australian Government, Department of Education and Training. AE acknowledges support from the Jack Brockhoff Foundation (JBF Grant number 4655–2019) and was supported by an Australian Research Council (ARC) Discovery Early Career Research Award (DECRA) (DE220100511). JT acknowledges support from the National Health and Medical Research Council of Australia (APP1181580). DG was supported by an NHMRC Senior Principal Research Fellowship (1117766) and an NHMRC Investigator Award (2008913), and acknowledges support from the National Health and Medical Research Council of Australia (NHMRC; 1113293). JT and DG acknowledge support from the Jack Ma Foundation for parts of the SARS-CoV-2 VLP work in this manuscript.

## Conflict of interest

VL, VP, and SM are employees of Sanofi-Pasteur. Part of the dengue VLP work was performed under an unrestricted research agreement between JT, University of Melbourne and Sanofi-Pasteur. There are two patents (PCT 35580344 and PCT 35580347) and three provisional patents (Patent Specification – 35541073, Patent Specification, SARS-CoV-2 VLP – 35555578, and Patent Specification, SARS-CoV-2 VLP – 35549058) covering this work. DG is a member of the Scientific Advisory Board of Avalia Immunotherapies. SG is a Director of Avalia Immunotherapies.

The remaining authors declare that the research was conducted in the absence of any commercial or financial relationships that could be construed as a potential conflict of interest.

## Publisher’s note

All claims expressed in this article are solely those of the authors and do not necessarily represent those of their affiliated organizations, or those of the publisher, the editors and the reviewers. Any product that may be evaluated in this article, or claim that may be made by its manufacturer, is not guaranteed or endorsed by the publisher.
