# Supplementary material for: Development of virus-like particles with inbuilt immunostimulatory properties as vaccine candidates
Source: Front Microbiol. 2023 Jun 7;14:1065609. doi: 10.3389/fmicb.2023.1065609 (PMC10282183; doi:10.3389/fmicb.2023.1065609)
Supplement: Supplementary file 1 [file Data_Sheet_1.docx]

Supplementary Material

Development of virus-like particles with inbuilt immunostimulatory properties as vaccine candidates

**Simon Collett^1^, Linda Earnest^2^, Julio Carrera Montoya^2^, Melissa A. Edeling^2^, Ashley Yap^2^, Chinn Yi Wong^2^, Dale Christiansen^2^, Jason Roberts^3,4^, Jamie Mumford^3^, Valerie Lecouturier^5^, Vincent Pavot^5^, Sergio Marco^5^, Joon Keit Loi^2^, Cameron Simmons^6^, Shivali A. Gulab^7,8^, Jason M. Mackenzie^2^, Aaron Elbourne^1^, Paul A. Ramsland^1,9,10^, Garth Cameron^2^, Dhiraj Hans^11^, Dale I. Godfrey^2^, Joseph Torresi^2*^**

^1^School of Science, College of Science, Engineering and Health, RMIT University, Melbourne, Victoria, Australia

^2^Department of Microbiology and Immunology, The Peter Doherty Institute for Infection and Immunity, University of Melbourne, Parkville, Victoria, Australia

^3^Victorian Infectious Diseases Reference laboratory, Royal Melbourne Hospital at the Doherty Institute for Infection and Immunity, Melbourne, Victoria, Australia.

^4^Department of Infectious Diseases, The University of Melbourne at the Doherty Institute for Infection and Immunity, Melbourne, Victoria, Australia.

^5^Sanofi-Pasteur, Lyon France

^6^Institute of Vector Borne Diseases, Monash University, Clayton, Victoria, Australia

^7^Avalia Immunotherapies Limited, Wellington, New Zealand

^8^Vaccine Alliance Aotearoa New Zealand, New Zealand

^9^Department of Surgery Austin Health, University of Melbourne, Heidelberg, Victoria, Australia

^10^Department of Immunology, Central Clinical School, Monash University, Melbourne, Victoria, Australia

^11^Research, Innovation & Commercialisation, Faculty of Medicine, Dentistry & Health Sciences, University of Melbourne, Parkville, Victoria, Australia

*** Correspondence:**Corresponding Author
josepht@unimelb.edu.au


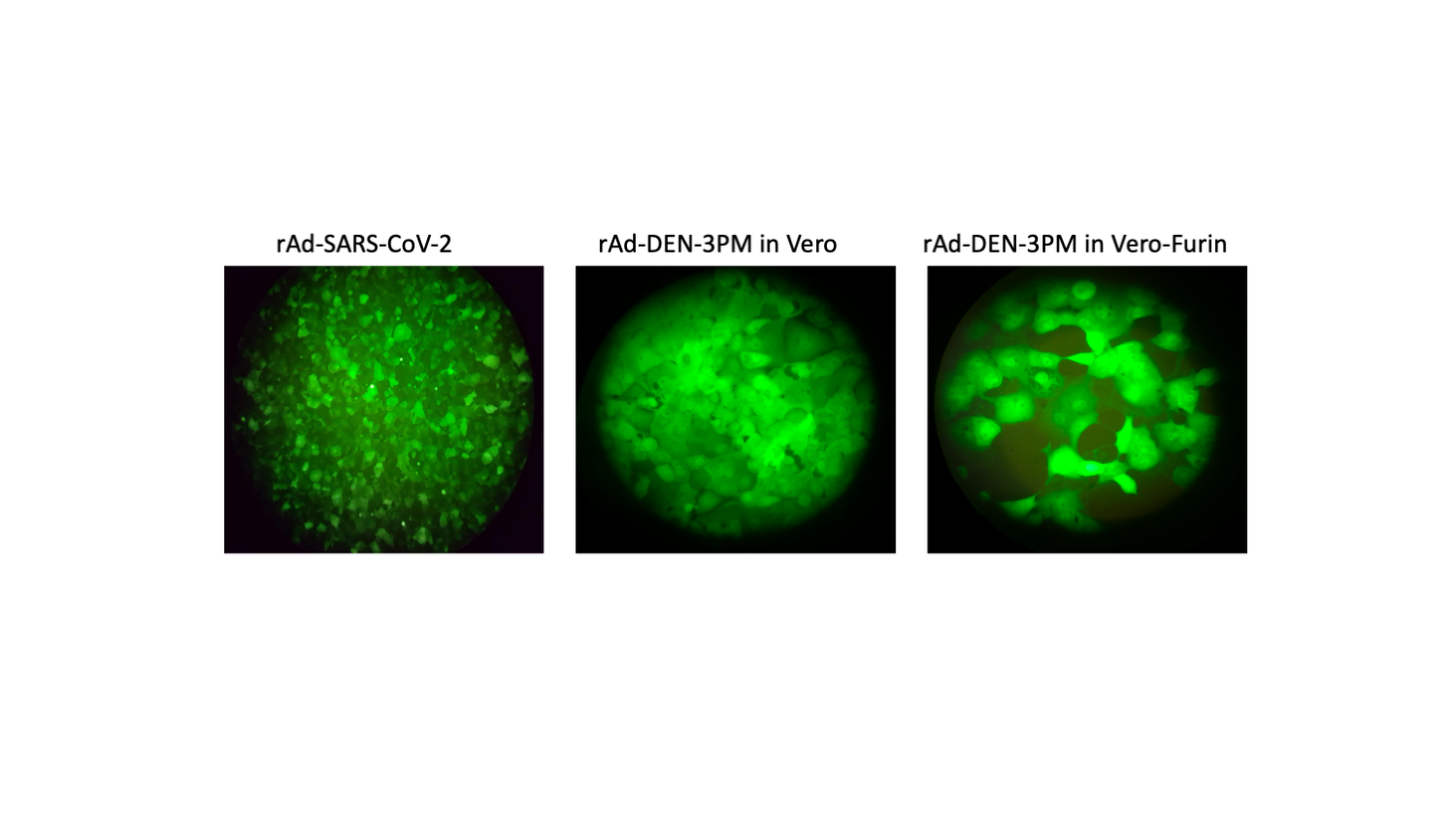


**SI Figure 1.** Infection of Vero and Vero-Furin cells with rAd-SARS-CoV-2 and rAd-DEN2core_HCV_-prM/E-3ptmut viruses. Cells were infected in 6-well tissue culture plates at a MOI of 1.0. After 72 hrs, plates were analysed by fluorescence microscopy and flow cytometry for GFP expression. Both viruses resulted in 90% transduction of cells.


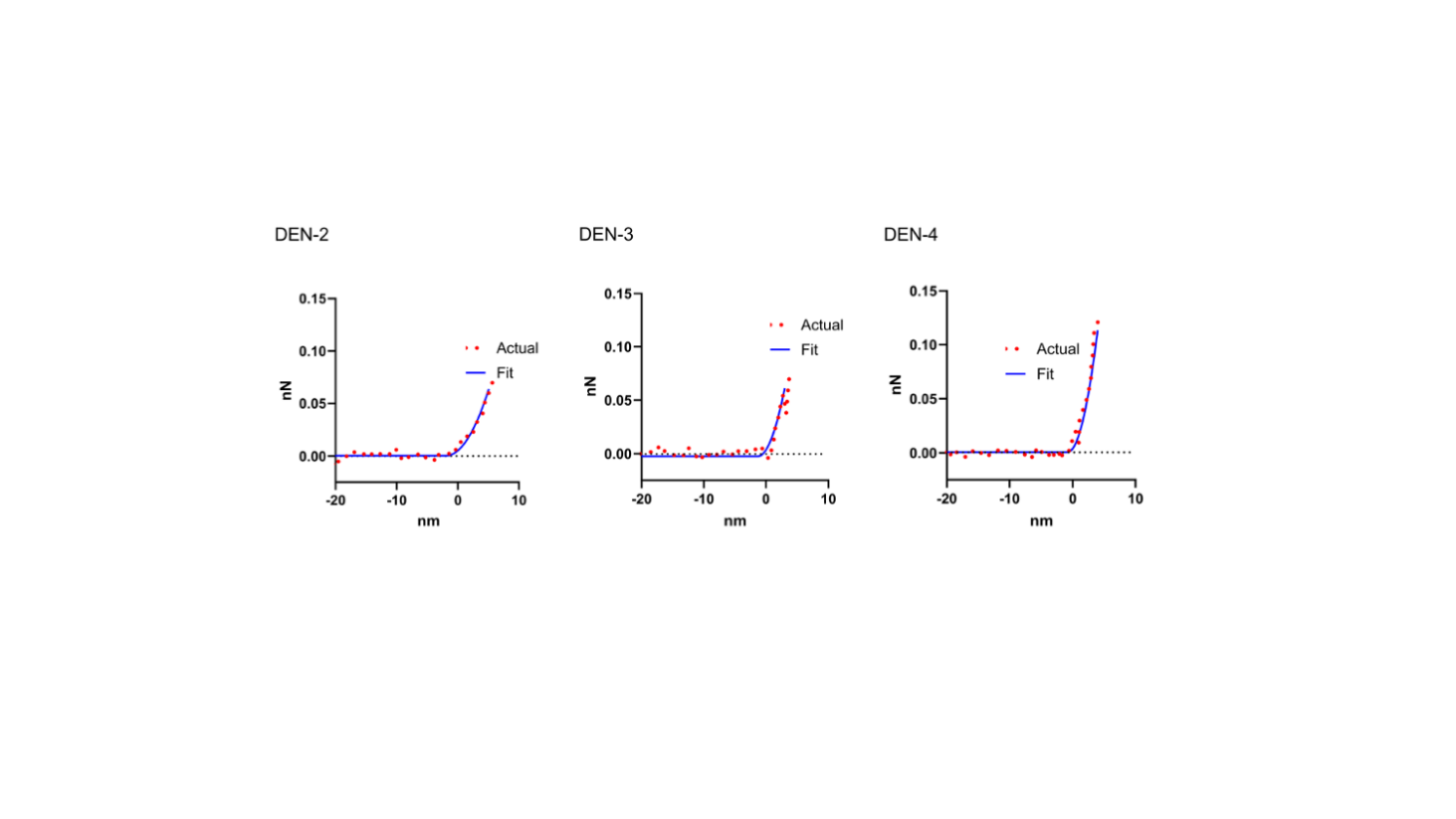


**SI Figure 2.** Representative force curves from each DEN VLP serotype fitted to the Sneddon/Hertz equation used to calculate Young’s elastic modulus.


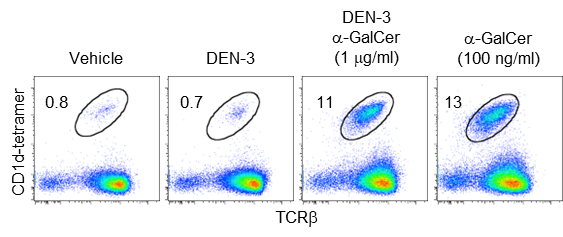


**SI Figure 3.** **NKT cell activation induced by self-adjuvanted DEN-3 VLPs.** Splenocytes (C57BL/6) were stimulated for 4 days *in vitro* under the indicated condition (VLPs 1 μg/ml) prior to FACS analysis. Numbers on plots indicate the percentage of NKT cells amongst viable (7-AAD-), CD19- lymphocytes.


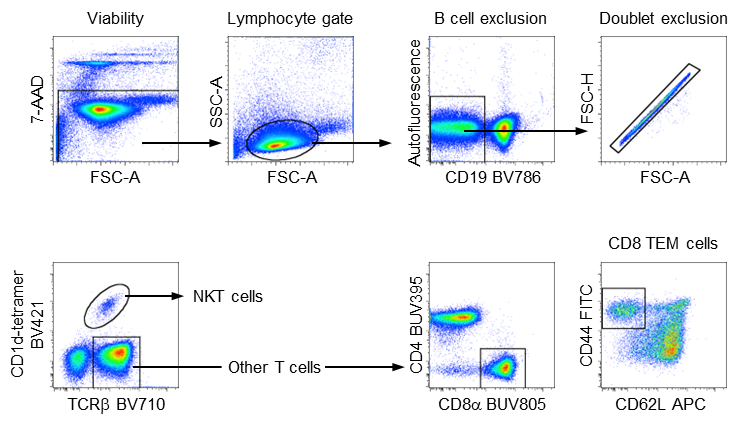


**SI Figure 4.** Flow cytometry gating strategy for identifying NKT and CD8 TEM cells.
